# Therapeutic interventions in children and adolescents with patellar tendon related pain: a systematic review

**DOI:** 10.1136/bmjsem-2018-000383

**Published:** 2018-08-13

**Authors:** George Cairns, Timothy Owen, Stefan Kluzek, Neal Thurley, Sinead Holden, Michael Skovdal Rathleff, Benjamin John Floyd Dean

**Affiliations:** 1 Bristol Medical School, University of Bristol Medical School, Bristol, UK; 2 Royal Free Hospital, Royal Free London NHS Foundation Trust, London, UK; 3 Nuffield Department of Orthopaedics, Rheumatology and Musculoskeletal Sciences (NDORMS), Botnar Research Centre, University of Oxford, Oxford, UK; 4 Nuffield Orthopaedic Centre, Oxford University Hospitals NHS Foundation Trust, Oxford, UK; 5 Bodleian Health Care Libraries, Cairns Library, John Radcliffe Hospital, Oxford, UK; 6 Department of Clinical Medicine, Aalborg University, Aalborg, Denmark; 7 Research Unit for General Practice in Aalborg, Department of Clinical Medicine, Aalborg University, Aalborg, Denmark

**Keywords:** tendinopathy, tendinosis, tendon, children, adolescent

## Abstract

**Objective:**

Evaluate effectiveness and harms of interventions for patellar tendon related pain in children and adolescents.

**Design:**

Systematic review and meta-analysis.

**Data sources:**

Medline via Pubmed, Embase via OVID, CINAHL via Ebsco, SportDiscus up until 24 November 2017 were searched.

**Eligibility criteria for selecting studies:**

Inclusion criteria were (1) controlled or randomised controlled clinical trials (RCTs), (2) participants with diagnosis of patellar tendon related disorder, (3) participants≤18 years of age at enrolment and (4) published in a peer-reviewed English or Scandinavian language journal.

**Results:**

Of 530 studies identified, eight were included after screening, with three included in data synthesis. To be included in data synthesis, we required studies to have included (and have data available for) a minimum of 10 participants under 18 years. All studies were rated as being at high risk of bias. For adolescents with patellar tendinopathy, one RCT compared eccentric exercises to usual care and found no difference between groups. In adolescents with Osgood-Schlatter disease (OSD), injection of local anaesthetic with dextrose proved superior to either usual care or local anaesthetic alone (three armed RCTs). In a retrospective case controlled study in adolescents with OSD, surgery provided no benefit over conservative management in terms of persistent symptoms and had a higher complication rate.

**Conclusion:**

There is weak evidence to support the use of dextrose injection with local anaesthetic and no evidence to support the use of specific types of exercises to treat children/adolescents with OSD/patellar tendinopathy. Until further evidence arises, clinicians should include load modification and advise on a return to sport based on symptoms.

**What is already known?**

- Patellar tendon related pain is relatively common in physically active children and adolescents.

**What are the new findings?**

- Only three randomised controlled clinical trials have tested an intervention versus comparator in a population with a reasonable number of children and adolescents.
- All studies had a high risk of bias. Specifically, ‘usual care’ provided was highly variable which provided a substantial source of bias.
- In one study, hyperosmolar dextrose injection (combined with local anaesthetic) was superior to both local anaesthetic injection and usual care.
- This review found no evidence to justify surgery for Osgood-Schlatter disease.

## Introduction

Patellar tendon related pain conditions are common in children and adolescents, appear to begin in childhood and increase in prevalence during adolescence up to age 18.^1^ Patellar tendon related pain is an umbrella term, which encompasses Osgood-Schlatter’s disease (OSD), Sinding-Larsen-Johansson disease and patellar tendinopathy. OSD affects 1 in 10 adolescents and as many as 1 in 5 in certain sports.^2 3^ There is a paucity of high-quality evidence relating to prognosis.

In one prospective cohort study in 18 adolescents with OSD, even 2 years after initial diagnosis, over 60% demonstrated persistent patellar tendon changes (evaluated by ultrasonography) and continued to display deficits in functional performance. Individuals with a history of OSD can experience persistent pain, years later, in their early 20s,^4–6^ associated with OSD lesions, underscoring that these pain complaints may not go away on their own if treated with a ‘wait and see’ approach. Interventions which reliably improve long-term outcomes are needed.^7^

While there is a large body of randomised trials and high-quality systematic reviews for managing patellar tendon related disorders in adults, there is a complete lack of systematic evaluations of treatment strategies specifically for adolescents and children.^8^ Considering patellar tendon related pain seem to start around the age of 10,^1^ there is a need for evidence and syntheses specific to this population. This is especially important considering some of these patellar tendon related pain complaints are unique to adolescent period, for example, Sinding-Larsen-Johansson syndrome and OSD.

The aim of this study was to perform a systematic review (and if possible, meta-analysis) of the benefits and harms of different treatment options for patella tendon related pain in children and adolescents.

## Methods

The systematic review was prospectively registered the review in PROSPERO (registration number 82736 link). The review was informed according to the Cochrane guidelines and is reported according to the PRISMA statement.

### Data sources and searches

We carried out a systematic search in the following bibliographic databases: Medline via Pubmed, Embase via OVID, CINAHL via Ebsco, SportDiscus up until 24 November 2017. The search terms and strategy were developed with a research librarian and are available in online supplementary material 1.

10.1136/bmjsem-2018-000383.supp1 Supplementary data

### Inclusion/exclusion criteria

Studies evaluating any intervention for any type of patellar tendon related pain in children or adolescents were eligible for inclusion, providing the design included an intervention and a comparator. Specifically, non-randomised controlled clinical trials and randomised controlled trials (RCTs) (including semi/quasi-randomised and cluster randomised trials) were eligible. Any therapeutic intervention or control treatments were included (including, but not restricted to, non-surgical interventions, injection-based interventions, exercise, surgical interventions or standard care/wait and see  and so on). Studies had to include children or adolescents (aged under 18 years), with patellar tendon related pain. We included any publications in English or Scandinavian languages. Studies were excluded if reporting on primary complaints of patellofemoral joint disorders including patellofemoral pain a, patellofemoral instability, acute traumatic causes of knee pain, inflammatory arthritis, or if they included a population age >18 years. If the study included a mixed-age population extending across the age limits in our inclusion criteria, we requested age-specific data for those <18 years from trial authors.

### Selection of studies

Duplicates were removed and relevant studies identified from the search were imported into Covidence for screening. Studies were independently screened by title and abstract by two authors (TO and GC). This was followed by full text evaluation of the selected studies from the first selection step by both authors. Disagreement between the two reviewers was solved by consensus involving a third reviewer (BD).

### Outcomes

Our primary domains of interest were pain, function and sport participation. Reports on the number and type(s) of adverse effects (harms) related to interventions were extracted and recorded. Additionally, all other reported outcomes were considered of potential interest and extracted. We did not impose any restrictions on endpoints; a priori we defined endpoints of interest as immediate effects (0–7 days after receiving treatment), short-term (1 week to 3 months), medium-term (3–6 months) and long-term (above 6 months).

### Data extraction

Two reviewers (GC and BD) independently extracted data. Data were extracted using a custom data extraction sheet in Covidence. Data regarding study design and setting, sample characteristics (including diagnoses, age, sex, demographics), inclusion criteria, intervention types and characteristics, follow-up, compliance, withdrawals, outcomes and any adverse events were extracted. Inconsistencies were resolved by consensus discussion. A third review (MSR) was available for disagreements that could not be resolved by discussion.

If relevant data were not available from full-text articles or trial registrations, authors were contacted to provide this information. For studies including mixed populations (both adolescents and adults), individual participant data for those 18 and under were requested. Authors were sent two subsequent reminders over 6 weeks. Studies were excluded from data synthesis if individual patients were not provided or studies included less than 10 participants under 18.

### Risk of bias assessment

Two independent raters (BD and GC) assessed risk of bias using the Cochrane Collaboration’s tool for assessing risk of bias in randomised trials. Each trial was evaluated across seven domains of bias, including one or more items that are were appraised in two parts. Assessment followed the description in the Cochrane Handbook for Systematic Review of Interventions, V.5.1 (Part 2: 8.5.1) as follows. First, the relevant trials’ characteristics related to the item were summarised. Second, each bias domain was judged as high or low risk of bias, according to their possible effect on the results of the trial. When the possible effect was unknown or insufficient detail was reported, the item was judged as unclear. Disagreements were resolved by discussion. A third party (MSR) was available in case of persistent disagreement.

### Data analysis

It was specified a priori, a meta-analysis would only be performed if data were available for similar time-points, outcomes and interventions. As this was not possible, we conducted a narrative synthesis of the results based on the domains of interest.

## Results

**Figure 1:**
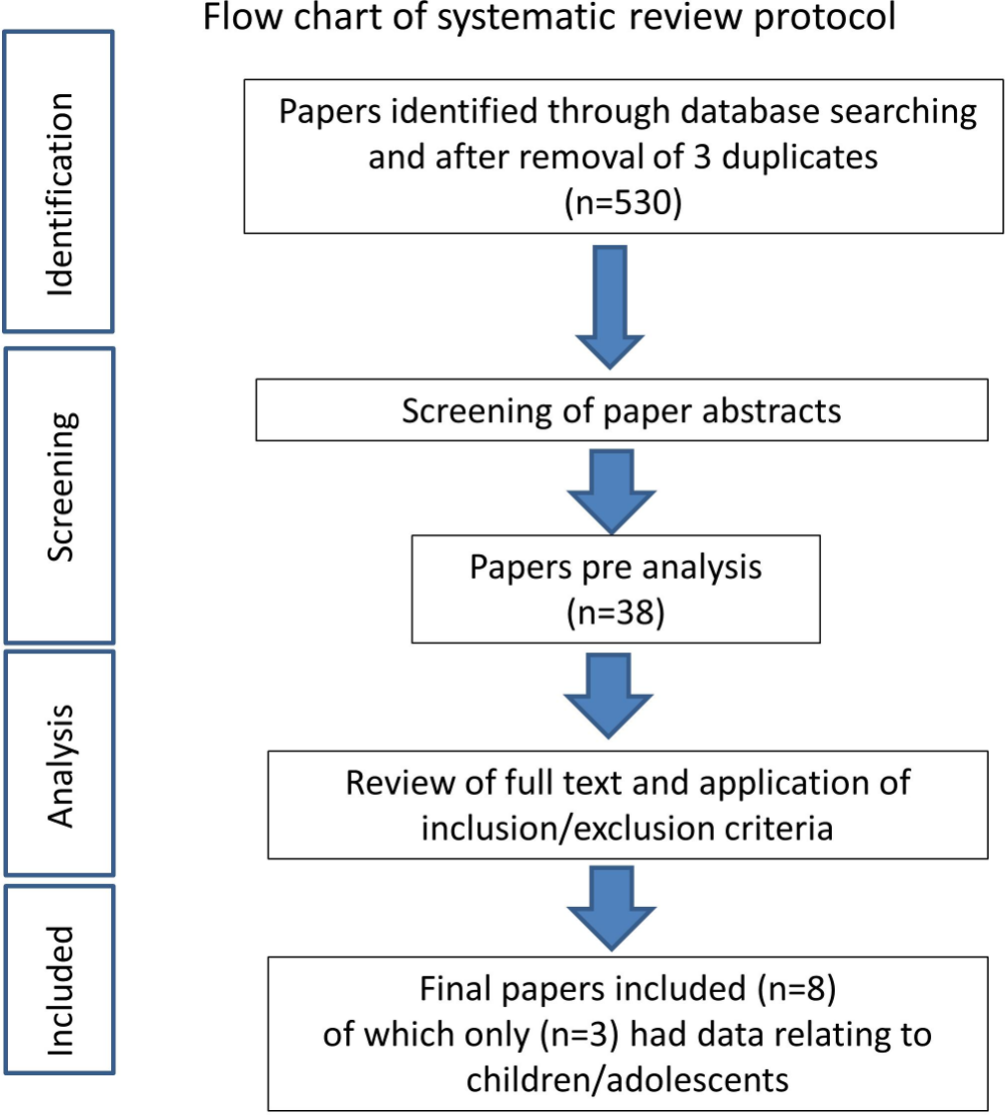
Flowchart of systematic review protocol.

A total of 530 studies were identified by the search strategy. After screening, eight studies were identified as eligible for inclusion (figure 1, PRISMA flowchart). Of these, seven were RCTs, and one was a retrospective non-randomised controlled trial. Two studies specifically included an adolescent-only population (both OSD). The other six studies were on patellar tendinopathy and included a mixed sample. Study characteristics are provided in table 1 and table 2. Additional information of included studies is available in online supplementary material 1 (S1).

10.1136/bmjsem-2018-000383.supp2 Supplementary data

**Table 1:** Study characteristics

Author	Year	Journal	Setting	Population	Age	Type of study	Intervention/s	Comparator	Outcomes	Time points
Biernat*et al* 11	2014	*J Strength* *Cond Res*	University physical therapy	Male volleyball players with patellar tendinopathy	Mixed	Randomised controlled trial	Specific exercises including eccentric decline squat	Continued competitive activity and functional exercises	Visa-P Maximal power Jump height	Baseline, 12 weeks, 24 weeks
Cannell*et al* 16	2001	*Br J Sports* *Med*	Sports medicine centre	Mixed athletes with jumper’s knee	Mixed	Randomised controlled trial	Drop squat exercises	Leg extension/curl exercises	Pain score Quadriceps moment Hamstring moment	Baseline, 4 weeks
Jonsson *et al* 14	2005	*Br J Sports* *Med*	Sports medicine unit	Athletes with jumper’s knee	Mixed	Randomised controlled trial	Eccentric exercises	Concentric exercises	VISA Pain scores (VAS)	Baseline, 6 weeks, 12 weeks
Topol*et al* 9	2011	*Pediatrics*	Physical medicine	Children with Osgood Schlatter’s who played sports	All <18 years old	Randomised controlled trial	Local anaesthetic and Local anaesthetic plus dextrose	Usual care	NPSS (Nirschl Pain Phase Scale)	Baseline, 3 months
Trail*et al* 10	1988	*J* *Pediatr Orthop*	Secondary care—Orthopaedics	Children with Osgood Schlatter’s	All <18 years old	Retrospective non-randomised parallel group design	Tibial sequestrectomy and mixture of cast/injections/physiotherapy	Reduction of activity/sport avoidance and mixture of cast/injections/physiotherapy	Proportion symptomatic Complication rate	Variable as at final review
Wang*et al* 12	2007	*Am* *J Sports Med*	Orthopaedic surgery department	Patients with chronic patellar tendinopathy	Mixed	Randomised controlled trial	Shockwave treatment followed by period of light activity	NSAIDs, physiotherapy and exercise program	Pain scores VISA Knee range of movement	Baseline, variable—2 to 3 years
Willberg*et al* 15	2011	*Br J Sports Med*	Sports medicine unit	Patients with patellar tendinopathy	Mixed	Randomised controlled trial	Arthroscopic shaving	Sclerosing polidocanol injections	VAS at rest VAS on activity Satisfaction	Baseline, variable—approximately 12 months
Van Ark *et al* 13	2016	*J Sci Med Sport*	Physiotherapy department	Volleyball/basketball players with patellar tendinopathy	Mixed	Randomised controlled trial	Isometric exercises	Isotonic exercises	Pain score	Baseline, 4 weeks

NSAIDs, non-steroidal anti-inflammatory drugs.

**Table 2:** Details of study participants demographics and whether data were provided

Author	Year	Journal	Intervention group age	Comparator group age	Intervention group sex	Comparator group sex
Biernat *et al* 11	2014	*J Strength Cond Res*	17.2 (0.6)	16.5 (0.8)	All male	All male
Cannell *et al* 16	2001	*Br J Sports Med*	No response from author within time limit
Jonsson *et al* 14	2005	*Br J Sports Med*	Author confirmed age specific data not obtainable
Topol, *et al* 9	2011	*P* *ediatrics*	13.3 (range 10–17)	51 boys and three girls
Trail *et al* 10	1988	*J Pediatr Orthop*	13 years 9 months (range 11–17)	12 years 7 months (range 10–17)	Not stated	Not stated
Wang *et al* 12	2007	*Am* *J Sports Med*	Author confirmed no participants under age 18 took part in the study (note study did state age range lower limit was 16)
Willberg *et al* 15	2011	*Br J Sports Med*	No response from author within time limit
Van Ark *et al* 13	2016	*J Sci Med Sport*	Only one under 18 year old participant took part hence study excluded from data analysis

### Osgood-Schlatter

Topol *et al* 9 compared usual care versus local anaesthetic versus local anaesthetic and dextrose in a threearmed RCT for OSD. The outcome was the Nirschl Pain Phase Scale (NPPS) at 3 months. The superiority of dextrose and local anaesthetic compared with local anaesthetic alone was shown (SMD: 0.83, 95% CI 0.20 to 1.45, p=0.006) or usual care (SMD: 1.66, 95% CI 0.96 to 2.36, p<0.0001) (see online supplementary material for Forest plots).

10.1136/bmjsem-2018-000383.supp3 Supplementary data

10.1136/bmjsem-2018-000383.supp4 Supplementary data

10.1136/bmjsem-2018-000383.supp5 Supplementary data

10.1136/bmjsem-2018-000383.supp6 Supplementary data

10.1136/bmjsem-2018-000383.supp7 Supplementary data

10.1136/bmjsem-2018-000383.supp8 Supplementary data

Trail^10^ compared surgery (tibial sequestrectomy) with conservative treatment in a retrospective non-randomised parallel group study of OSD. Participants were categorically rated as ‘symptomatic’ or ‘asymptomatic’ at final review approximately 5 years from initial diagnosis. The odds of being asymptomatic was not significantly different with surgery compared with conservative treatment (OR 1.48, 95% CI 0.33 to 6.65) (see online supplementary material for Forest plots). Several patients in both groups were subject to other interventions (including refraining from sport, cast treatment, physiotherapy and steroid injections).

### Patellar tendinopathy

Only one^11^ of the six studies including mixed populations of adolescents and adults was included in the data synthesis (details of exclusion outlined below). This study by Biernat *et al* compared the effect of usual care to eccentric exercises in male adolescents on patient-recorded outcomes (VISA-P questionnaire). There was no significant difference between groups at either 12 or 24 weeks (SMD: 0.45, 95% CI −0.44 to 1.35, p=0.32 and SMD 0.37, 95% CI 0.52 to 1.26, p=0.42, respectively) (see online supplementary material for Forest plot) table 2.

Two studies were excluded from data synthesis as they did not contain a large enough sample of adolescents (N=0 and N=1 adolescent included).^12 13^ One study was excluded from data synthesis because data for adolescents could not be extracted,^14^ and two studies could not be included in the data synthesis due to non-response to requests for data for adolescent participants.^15 16^

### Harms

Only one study reported on potential harms. Trail reported a lower overall complication rate for conservative treatment,^10^ compared with surgery (OR 0.11, 95% CI 0.03 to 0.38) (see online supplementary material for plot  harms OR).^10^ Complications were primarily presence of bony prominence (6 in conservative group and 18 in surgical group). The surgical group had additional complications (three with areas of anaesthesia lateral to scar, one infection, one wound dehiscence, one stiffness and one recurvatum).

### Risk of bias

**Figure 2:**
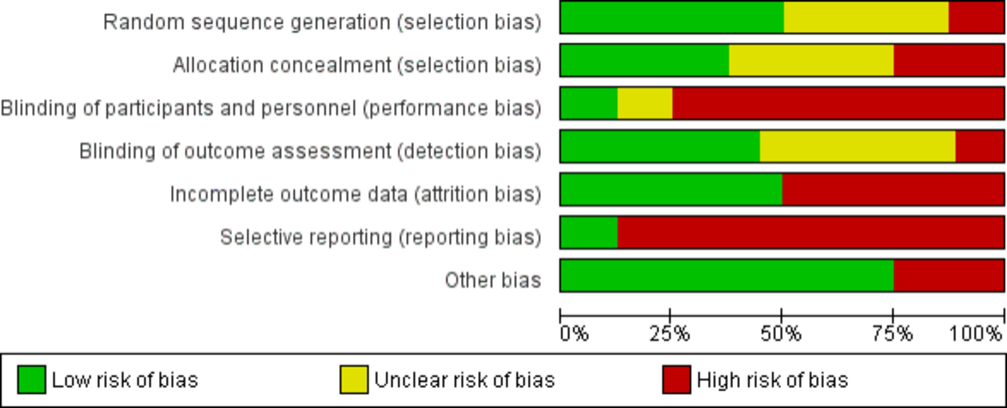
Risk of bias within studies.

**Figure 3:**
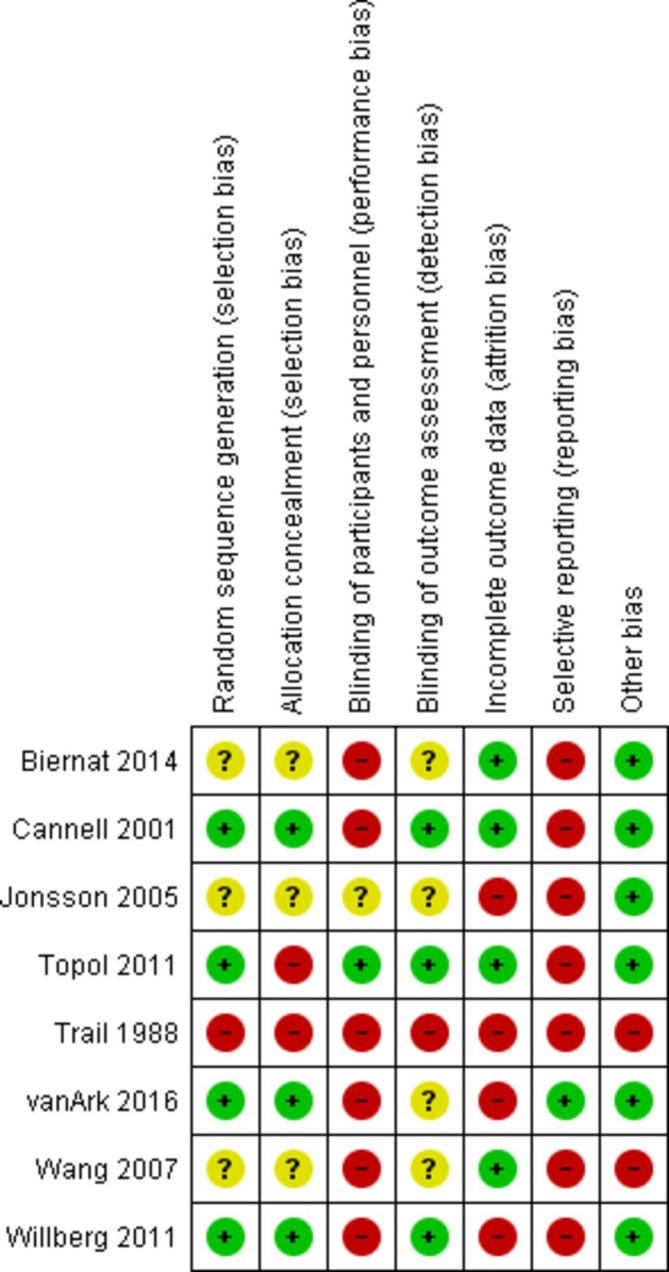
Risk of bias summary graph.

Overall, all studies were deemed to be at high risk of bias, particularly in the domains of reporting bias and performance bias (figure 2 and figure 3)

Studies identified and excluded at each stage are detailed in figure 1.

## Discussion

The key finding of this review is the lack of studies assessing the effectiveness of interventions for patellar tendon related pain in children and adolescents. Despite commonality of these disorders in this population, only three studies have assessed any intervention. All three were at high risk of bias, especially reporting bias, blinding and had significant methodological weaknesses (with one being a retrospective parallel group case-series). This makes in nearly impossible to guide clinical practice on how to best to manage these common pain complaints in children and adolescents.

### Adults and adolescents—potentially not the same

The problem is that while there is evidence supporting certain interventions (eg, tendon loading programmes) for patellar tendon related pain in adults,^17^ the findings of these cannot be directly transferred into children and adolescents. The pathological features of tendinopathy includes increased cellularity, a loss of collagen structure and chondroid metaplasia.^18 19^ There is an increasing body of evidence demonstration the role of chronic inflammation as part of this degenerative process^18 20 21^ Tendon loading programmes have been associated with an improved collagen turnover in adults suggesting a possible mechanisms of effect. However, an important difference between adults and adolescents is the maturity of the patellar tendon insertion, the enthesis organ.^22^ It is a possible that overly aggressive sporting activity and tendon loading during development may actually have a negative long-term impact in terms of both tendon structure and the risk of adulthood symptoms. Notably, a recent study has shown that increased sporting activity has a detrimental impact on the developing hip, which supports this theory. As we do not yet have a clear understanding of how the pathology of patellar tendon related pain during maturation, caution needs to be taken in applying such effective interventions from adults to children and adolescents. This is particularly as adolescence is proposed to be a critical time for the formation of normal tendon attachment.^23 24^

### Devil in the detail

One of the serious concerns with the included studies is the multiple differences in treatment between control and experimental arms. For example, in Topol *et al* 9 the control group did not have a period of rest, which was in contrast to both injection therapy groups. This makes it difficult to elucidate if the observed effects were actually due to the intervention or to the removal of aggravating activities. Furthermore, a dextrose injection at 3 months was used as an ‘incentive’ for study participation and offered to all participants that did not reach an NPPS score of 0 (regardless of treatment allocation), to avoid dropout in the control arm. This could potentially influence participants’ reporting, if they had a favourable perception of the injection, and reported lower outcomes to receive it.

The high frequency of patellar tendon related disorders in the sporting population is consistent with an ’overuse’ type aetiology. In this context, any advice on activity modification and the manner in which it is delivered may be crucial in influencing both short-term and long-term outcomes. Keeping this in mind, it is arguable what constitutes ‘usual care’ in daily practice and in the included studies may have been. The ‘usual care’ was highly variable and included factors such as the degree to which sporting activity was continued and the degree to which pain was experienced and/or allowed during sport or exercises. In the study by Topol *et al*,^9^ only participants in the injection arms were encouraged to engage in a sporting activity, only if the activity was not accompanied or followed by pain during the period of treatment. This contrasted with Biernat *et al* in which both experimental and control groups were subject to the same advice as regards continuing sporting activity.^11^

### Harms

The study by Trail^10^ demonstrated a significantly higher complication rate with surgery versus conservative treatment, indicating that currently the use of surgery in this context should only take place in the more regulated context of a clinical trial. Notably, no studies analysed longer term outcomes into early adulthood, highlighting an area for future research.

### Lack of knowledge hampers clinical practice

The absence of high-quality evidence presents a challenge to clinicians treating children and adolescents with patellar tendon related pain. Adolescence is a key developmental period, with a mature proximal patellar tendon enthesis occurring after peak height velocity.^25^ Given that patellar tendon structure is a predictor for pain,^26^ it is reasonable to argue that caution should be exercised when treating patients with an immature enthesis. Training beyond pain in order to satisfy short-term sporting objectives could have negative long term implications for the individual.^27^ Until further evidence arises, clinicians should be aware of the potential differences in patellar tendon related pain between adolescents and adults. These types of pain complaints are often associated with high levels of sports participation and sports specialisation. As sustained overload is thought to be a risk factor for developing, for example, OSD,^28^ it seems sensible that the mainstay of treatment include some form of load management. This type of treatment could include education where adolescents and parents are taught to modify training and loading (including recovery) based on symptoms but this has yet to be evaluated in a clinical trial.

### Future research

Malliaras *et al* 17 concluded that high-quality studies comparing different loading programmes and evaluating clinical and mechanistic outcomes are needed in both Achilles and patellar tendinopathy rehabilitation in adults.^17^ The evidence relating to adolescents and children is worse, and this is concerning given the potential long-term consequences of overuse on the developing bone tendon interface. Understanding the impact of patellar tendon related pain disorders on the maturing tendon and entheses on both structural and functional outcomes in both the short and longer term, will be critical in the understanding of the natural history of patellar tendon related disorders in adolescents. It is also essential to explore the needs and preference of the young patients being treated. It is likely that these developing young individuals have specific needs which have yet to be addressed during treatment. Future studies may also wish to identify phenotypes associated with a particular poor prognosis to understand the trajectory of pain and impairments during this crucial period of life.

Despite the commonality of patella tendon related pain in children and adolescents, there is a paucity of evidence specific to this population to guide clinical practice. There is weak evidence to support the use of dextrose injections and no evidence to support the use of specific types of exercises. Further research is essential to ascertain how best to manage the many children and adolescents suffering from these pain complaints. Until further evidence arises, clinicians should include load modification and advise on a return to sport based on symptoms.
