# Supplementary material for: Therapeutic interventions in children and adolescents with patellar tendon related pain: a systematic review
Source: BMJ Open Sport Exerc Med. 2018 Aug 13;4(1):e000383. doi: 10.1136/bmjsem-2018-000383 (PMC6109948; doi:10.1136/bmjsem-2018-000383)
Supplement: Supplementary data [file bmjsem-2018-000383supp001.docx]

**MEDLINE: (24/11/17)**

| \| \| **Searches** \| **Results** \| **Type** \| **Actions** \| **Annotations** \| \| --- \| --- \| --- \| --- \| --- \| \|  \| \| \| \| \| \| \| \|  \| 1 \| Patella/ \| 10157 \| Advanced \| [Display Results](http://ovidsp.tx.ovid.com/sp-3.27.1a/ovidweb.cgi?&S=DPDPFPJMBHDDHOGKNCFKKEIBELHNAA00&SELECT=S.sh%7c&R=1&Process+Action=display)  [More](http://ovidsp.tx.ovid.com/sp-3.27.1a/ovidweb.cgi?&S=DPDPFPJMBHDDHOGKNCFKKEIBELHNAA00&SELECT=S.sh%7c&Expand=1&Main+Search+Page=Main+Search+Page) \| [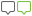](http://ovidsp.tx.ovid.com/sp-3.27.1a/ovidweb.cgi?&S=DPDPFPJMBHDDHOGKNCFKKEIBELHNAA00&R=1&Search+Annotations+Options=SA) \| \|  \| 2 \| Tendinopathy/ \| 5690 \| Advanced \| [Display Results](http://ovidsp.tx.ovid.com/sp-3.27.1a/ovidweb.cgi?&S=DPDPFPJMBHDDHOGKNCFKKEIBELHNAA00&SELECT=S.sh%7c&R=2&Process+Action=display)  [More](http://ovidsp.tx.ovid.com/sp-3.27.1a/ovidweb.cgi?&S=DPDPFPJMBHDDHOGKNCFKKEIBELHNAA00&SELECT=S.sh%7c&Expand=1&Main+Search+Page=Main+Search+Page) \| [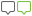](http://ovidsp.tx.ovid.com/sp-3.27.1a/ovidweb.cgi?&S=DPDPFPJMBHDDHOGKNCFKKEIBELHNAA00&R=2&Search+Annotations+Options=SA) \| \|  \| 3 \| 1 and 2 \| 160 \| Advanced \| [Display Results](http://ovidsp.tx.ovid.com/sp-3.27.1a/ovidweb.cgi?&S=DPDPFPJMBHDDHOGKNCFKKEIBELHNAA00&SELECT=S.sh%7c&R=3&Process+Action=display)  [More](http://ovidsp.tx.ovid.com/sp-3.27.1a/ovidweb.cgi?&S=DPDPFPJMBHDDHOGKNCFKKEIBELHNAA00&SELECT=S.sh%7c&Expand=1&Main+Search+Page=Main+Search+Page) \| [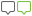](http://ovidsp.tx.ovid.com/sp-3.27.1a/ovidweb.cgi?&S=DPDPFPJMBHDDHOGKNCFKKEIBELHNAA00&R=3&Search+Annotations+Options=SA) \| \|  \| 4 \| ((patella* or kneecap* or knee cap*) adj3 (tendon* or tendin*)).ti,ab. \| 6097 \| Advanced \| [Display Results](http://ovidsp.tx.ovid.com/sp-3.27.1a/ovidweb.cgi?&S=DPDPFPJMBHDDHOGKNCFKKEIBELHNAA00&SELECT=S.sh%7c&R=4&Process+Action=display)  [More](http://ovidsp.tx.ovid.com/sp-3.27.1a/ovidweb.cgi?&S=DPDPFPJMBHDDHOGKNCFKKEIBELHNAA00&SELECT=S.sh%7c&Expand=1&Main+Search+Page=Main+Search+Page) \| [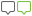](http://ovidsp.tx.ovid.com/sp-3.27.1a/ovidweb.cgi?&S=DPDPFPJMBHDDHOGKNCFKKEIBELHNAA00&R=4&Search+Annotations+Options=SA) \| \|  \| 5 \| Patellar Ligament/ \| 2449 \| Advanced \| [Display Results](http://ovidsp.tx.ovid.com/sp-3.27.1a/ovidweb.cgi?&S=DPDPFPJMBHDDHOGKNCFKKEIBELHNAA00&SELECT=S.sh%7c&R=5&Process+Action=display)  [More](http://ovidsp.tx.ovid.com/sp-3.27.1a/ovidweb.cgi?&S=DPDPFPJMBHDDHOGKNCFKKEIBELHNAA00&SELECT=S.sh%7c&Expand=1&Main+Search+Page=Main+Search+Page) \| [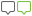](http://ovidsp.tx.ovid.com/sp-3.27.1a/ovidweb.cgi?&S=DPDPFPJMBHDDHOGKNCFKKEIBELHNAA00&R=5&Search+Annotations+Options=SA) \| \|  \| 6 \| Tendon Injuries/ \| 13267 \| Advanced \| [Display Results](http://ovidsp.tx.ovid.com/sp-3.27.1a/ovidweb.cgi?&S=DPDPFPJMBHDDHOGKNCFKKEIBELHNAA00&SELECT=S.sh%7c&R=6&Process+Action=display)  [More](http://ovidsp.tx.ovid.com/sp-3.27.1a/ovidweb.cgi?&S=DPDPFPJMBHDDHOGKNCFKKEIBELHNAA00&SELECT=S.sh%7c&Expand=1&Main+Search+Page=Main+Search+Page) \| [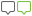](http://ovidsp.tx.ovid.com/sp-3.27.1a/ovidweb.cgi?&S=DPDPFPJMBHDDHOGKNCFKKEIBELHNAA00&R=6&Search+Annotations+Options=SA) \| \|  \| 7 \| 1 and 6 \| 338 \| Advanced \| [Display Results](http://ovidsp.tx.ovid.com/sp-3.27.1a/ovidweb.cgi?&S=DPDPFPJMBHDDHOGKNCFKKEIBELHNAA00&SELECT=S.sh%7c&R=7&Process+Action=display)  [More](http://ovidsp.tx.ovid.com/sp-3.27.1a/ovidweb.cgi?&S=DPDPFPJMBHDDHOGKNCFKKEIBELHNAA00&SELECT=S.sh%7c&Expand=1&Main+Search+Page=Main+Search+Page) \| [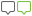](http://ovidsp.tx.ovid.com/sp-3.27.1a/ovidweb.cgi?&S=DPDPFPJMBHDDHOGKNCFKKEIBELHNAA00&R=7&Search+Annotations+Options=SA) \| \|  \| 8 \| osgood schlatter*.ti,ab. \| 344 \| Advanced \| [Display Results](http://ovidsp.tx.ovid.com/sp-3.27.1a/ovidweb.cgi?&S=DPDPFPJMBHDDHOGKNCFKKEIBELHNAA00&SELECT=S.sh%7c&R=8&Process+Action=display)  [More](http://ovidsp.tx.ovid.com/sp-3.27.1a/ovidweb.cgi?&S=DPDPFPJMBHDDHOGKNCFKKEIBELHNAA00&SELECT=S.sh%7c&Expand=1&Main+Search+Page=Main+Search+Page) \| [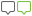](http://ovidsp.tx.ovid.com/sp-3.27.1a/ovidweb.cgi?&S=DPDPFPJMBHDDHOGKNCFKKEIBELHNAA00&R=8&Search+Annotations+Options=SA) \| \|  \| 9 \| sinding larsen johansson*.ti,ab. \| 41 \| Advanced \| [Display Results](http://ovidsp.tx.ovid.com/sp-3.27.1a/ovidweb.cgi?&S=DPDPFPJMBHDDHOGKNCFKKEIBELHNAA00&SELECT=S.sh%7c&R=9&Process+Action=display)  [More](http://ovidsp.tx.ovid.com/sp-3.27.1a/ovidweb.cgi?&S=DPDPFPJMBHDDHOGKNCFKKEIBELHNAA00&SELECT=S.sh%7c&Expand=1&Main+Search+Page=Main+Search+Page) \| [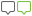](http://ovidsp.tx.ovid.com/sp-3.27.1a/ovidweb.cgi?&S=DPDPFPJMBHDDHOGKNCFKKEIBELHNAA00&R=9&Search+Annotations+Options=SA) \| \|  \| 10 \| jumpers knee*.ti,ab. \| 210 \| Advanced \| [Display Results](http://ovidsp.tx.ovid.com/sp-3.27.1a/ovidweb.cgi?&S=DPDPFPJMBHDDHOGKNCFKKEIBELHNAA00&SELECT=S.sh%7c&R=10&Process+Action=display)  [More](http://ovidsp.tx.ovid.com/sp-3.27.1a/ovidweb.cgi?&S=DPDPFPJMBHDDHOGKNCFKKEIBELHNAA00&SELECT=S.sh%7c&Expand=1&Main+Search+Page=Main+Search+Page) \| [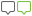](http://ovidsp.tx.ovid.com/sp-3.27.1a/ovidweb.cgi?&S=DPDPFPJMBHDDHOGKNCFKKEIBELHNAA00&R=10&Search+Annotations+Options=SA) \| \|  \| 11 \| (apophy* adj3 tibia* tuber*).ti,ab. \| 32 \| Advanced \| [Display Results](http://ovidsp.tx.ovid.com/sp-3.27.1a/ovidweb.cgi?&S=DPDPFPJMBHDDHOGKNCFKKEIBELHNAA00&SELECT=S.sh%7c&R=11&Process+Action=display)  [More](http://ovidsp.tx.ovid.com/sp-3.27.1a/ovidweb.cgi?&S=DPDPFPJMBHDDHOGKNCFKKEIBELHNAA00&SELECT=S.sh%7c&Expand=1&Main+Search+Page=Main+Search+Page) \| [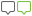](http://ovidsp.tx.ovid.com/sp-3.27.1a/ovidweb.cgi?&S=DPDPFPJMBHDDHOGKNCFKKEIBELHNAA00&R=11&Search+Annotations+Options=SA) \| \|  \| 12 \| lannelongue*.ti,ab. \| 60 \| Advanced \| [Display Results](http://ovidsp.tx.ovid.com/sp-3.27.1a/ovidweb.cgi?&S=DPDPFPJMBHDDHOGKNCFKKEIBELHNAA00&SELECT=S.sh%7c&R=12&Process+Action=display)  [More](http://ovidsp.tx.ovid.com/sp-3.27.1a/ovidweb.cgi?&S=DPDPFPJMBHDDHOGKNCFKKEIBELHNAA00&SELECT=S.sh%7c&Expand=1&Main+Search+Page=Main+Search+Page) \| [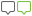](http://ovidsp.tx.ovid.com/sp-3.27.1a/ovidweb.cgi?&S=DPDPFPJMBHDDHOGKNCFKKEIBELHNAA00&R=12&Search+Annotations+Options=SA) \| \|  \| 13 \| (lannelongue* adj3 (disease* or syndrom* or condition* or disorder*)).ti,ab. \| 1 \| Advanced \| [Display Results](http://ovidsp.tx.ovid.com/sp-3.27.1a/ovidweb.cgi?&S=DPDPFPJMBHDDHOGKNCFKKEIBELHNAA00&SELECT=S.sh%7c&R=13&Process+Action=display)  [More](http://ovidsp.tx.ovid.com/sp-3.27.1a/ovidweb.cgi?&S=DPDPFPJMBHDDHOGKNCFKKEIBELHNAA00&SELECT=S.sh%7c&Expand=1&Main+Search+Page=Main+Search+Page) \| [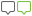](http://ovidsp.tx.ovid.com/sp-3.27.1a/ovidweb.cgi?&S=DPDPFPJMBHDDHOGKNCFKKEIBELHNAA00&R=13&Search+Annotations+Options=SA) \| \|  \| 14 \| 3 or 4 or 5 or 7 or 8 or 9 or 10 or 11 or 12 \| 7431 \| Advanced \| [Display Results](http://ovidsp.tx.ovid.com/sp-3.27.1a/ovidweb.cgi?&S=DPDPFPJMBHDDHOGKNCFKKEIBELHNAA00&SELECT=S.sh%7c&R=14&Process+Action=display)  [More](http://ovidsp.tx.ovid.com/sp-3.27.1a/ovidweb.cgi?&S=DPDPFPJMBHDDHOGKNCFKKEIBELHNAA00&SELECT=S.sh%7c&Expand=1&Main+Search+Page=Main+Search+Page) \| [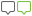](http://ovidsp.tx.ovid.com/sp-3.27.1a/ovidweb.cgi?&S=DPDPFPJMBHDDHOGKNCFKKEIBELHNAA00&R=14&Search+Annotations+Options=SA) \| \|  \| 15 \| exp Pediatrics/ \| 55872 \| Advanced \| [Display Results](http://ovidsp.tx.ovid.com/sp-3.27.1a/ovidweb.cgi?&S=DPDPFPJMBHDDHOGKNCFKKEIBELHNAA00&SELECT=S.sh%7c&R=15&Process+Action=display)  [More](http://ovidsp.tx.ovid.com/sp-3.27.1a/ovidweb.cgi?&S=DPDPFPJMBHDDHOGKNCFKKEIBELHNAA00&SELECT=S.sh%7c&Expand=1&Main+Search+Page=Main+Search+Page) \| [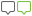](http://ovidsp.tx.ovid.com/sp-3.27.1a/ovidweb.cgi?&S=DPDPFPJMBHDDHOGKNCFKKEIBELHNAA00&R=15&Search+Annotations+Options=SA) \| \|  \| 16 \| exp Child/ \| 1880489 \| Advanced \| [Display Results](http://ovidsp.tx.ovid.com/sp-3.27.1a/ovidweb.cgi?&S=DPDPFPJMBHDDHOGKNCFKKEIBELHNAA00&SELECT=S.sh%7c&R=16&Process+Action=display)  [More](http://ovidsp.tx.ovid.com/sp-3.27.1a/ovidweb.cgi?&S=DPDPFPJMBHDDHOGKNCFKKEIBELHNAA00&SELECT=S.sh%7c&Expand=1&Main+Search+Page=Main+Search+Page) \| [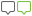](http://ovidsp.tx.ovid.com/sp-3.27.1a/ovidweb.cgi?&S=DPDPFPJMBHDDHOGKNCFKKEIBELHNAA00&R=16&Search+Annotations+Options=SA) \| \|  \| 17 \| exp Infant/ \| 1131298 \| Advanced \| [Display Results](http://ovidsp.tx.ovid.com/sp-3.27.1a/ovidweb.cgi?&S=DPDPFPJMBHDDHOGKNCFKKEIBELHNAA00&SELECT=S.sh%7c&R=17&Process+Action=display)  [More](http://ovidsp.tx.ovid.com/sp-3.27.1a/ovidweb.cgi?&S=DPDPFPJMBHDDHOGKNCFKKEIBELHNAA00&SELECT=S.sh%7c&Expand=1&Main+Search+Page=Main+Search+Page) \| [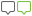](http://ovidsp.tx.ovid.com/sp-3.27.1a/ovidweb.cgi?&S=DPDPFPJMBHDDHOGKNCFKKEIBELHNAA00&R=17&Search+Annotations+Options=SA) \| \|  \| 18 \| Adolescent/ \| 1982178 \| Advanced \| [Display Results](http://ovidsp.tx.ovid.com/sp-3.27.1a/ovidweb.cgi?&S=DPDPFPJMBHDDHOGKNCFKKEIBELHNAA00&SELECT=S.sh%7c&R=18&Process+Action=display)  [More](http://ovidsp.tx.ovid.com/sp-3.27.1a/ovidweb.cgi?&S=DPDPFPJMBHDDHOGKNCFKKEIBELHNAA00&SELECT=S.sh%7c&Expand=1&Main+Search+Page=Main+Search+Page) \| [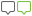](http://ovidsp.tx.ovid.com/sp-3.27.1a/ovidweb.cgi?&S=DPDPFPJMBHDDHOGKNCFKKEIBELHNAA00&R=18&Search+Annotations+Options=SA) \| \|  \| 19 \| (child* or infant* or paediatric* or pediatric* or adolescen* or teen* or youth* or boy* or girl* neonat* or baby or babies or babys).ti,ab. \| 1956829 \| Advanced \| [Display Results](http://ovidsp.tx.ovid.com/sp-3.27.1a/ovidweb.cgi?&S=DPDPFPJMBHDDHOGKNCFKKEIBELHNAA00&SELECT=S.sh%7c&R=19&Process+Action=display)  [More](http://ovidsp.tx.ovid.com/sp-3.27.1a/ovidweb.cgi?&S=DPDPFPJMBHDDHOGKNCFKKEIBELHNAA00&SELECT=S.sh%7c&Expand=1&Main+Search+Page=Main+Search+Page) \| [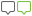](http://ovidsp.tx.ovid.com/sp-3.27.1a/ovidweb.cgi?&S=DPDPFPJMBHDDHOGKNCFKKEIBELHNAA00&R=19&Search+Annotations+Options=SA) \| \|  \| 20 \| 15 or 16 or 17 or 18 or 19 \| 3980454 \| Advanced \| [Display Results](http://ovidsp.tx.ovid.com/sp-3.27.1a/ovidweb.cgi?&S=DPDPFPJMBHDDHOGKNCFKKEIBELHNAA00&SELECT=S.sh%7c&R=20&Process+Action=display)  [More](http://ovidsp.tx.ovid.com/sp-3.27.1a/ovidweb.cgi?&S=DPDPFPJMBHDDHOGKNCFKKEIBELHNAA00&SELECT=S.sh%7c&Expand=1&Main+Search+Page=Main+Search+Page) \| [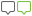](http://ovidsp.tx.ovid.com/sp-3.27.1a/ovidweb.cgi?&S=DPDPFPJMBHDDHOGKNCFKKEIBELHNAA00&R=20&Search+Annotations+Options=SA) \| \|  \| 21 \| 14 and 20 \| 2002 \| Advanced \| [Display Results](http://ovidsp.tx.ovid.com/sp-3.27.1a/ovidweb.cgi?&S=DPDPFPJMBHDDHOGKNCFKKEIBELHNAA00&SELECT=S.sh%7c&R=21&Process+Action=display)  [More](http://ovidsp.tx.ovid.com/sp-3.27.1a/ovidweb.cgi?&S=DPDPFPJMBHDDHOGKNCFKKEIBELHNAA00&SELECT=S.sh%7c&Expand=1&Main+Search+Page=Main+Search+Page) \| [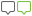](http://ovidsp.tx.ovid.com/sp-3.27.1a/ovidweb.cgi?&S=DPDPFPJMBHDDHOGKNCFKKEIBELHNAA00&R=21&Search+Annotations+Options=SA) \| \|  \| 22 \| randomized controlled trial.pt. \| 505458 \| Advanced \| [Display Results](http://ovidsp.tx.ovid.com/sp-3.27.1a/ovidweb.cgi?&S=DPDPFPJMBHDDHOGKNCFKKEIBELHNAA00&SELECT=S.sh%7c&R=22&Process+Action=display)  [More](http://ovidsp.tx.ovid.com/sp-3.27.1a/ovidweb.cgi?&S=DPDPFPJMBHDDHOGKNCFKKEIBELHNAA00&SELECT=S.sh%7c&Expand=1&Main+Search+Page=Main+Search+Page) \| [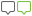](http://ovidsp.tx.ovid.com/sp-3.27.1a/ovidweb.cgi?&S=DPDPFPJMBHDDHOGKNCFKKEIBELHNAA00&R=22&Search+Annotations+Options=SA) \| \|  \| 23 \| controlled clinical trial.pt. \| 100426 \| Advanced \| [Display Results](http://ovidsp.tx.ovid.com/sp-3.27.1a/ovidweb.cgi?&S=DPDPFPJMBHDDHOGKNCFKKEIBELHNAA00&SELECT=S.sh%7c&R=23&Process+Action=display)  [More](http://ovidsp.tx.ovid.com/sp-3.27.1a/ovidweb.cgi?&S=DPDPFPJMBHDDHOGKNCFKKEIBELHNAA00&SELECT=S.sh%7c&Expand=1&Main+Search+Page=Main+Search+Page) \| [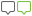](http://ovidsp.tx.ovid.com/sp-3.27.1a/ovidweb.cgi?&S=DPDPFPJMBHDDHOGKNCFKKEIBELHNAA00&R=23&Search+Annotations+Options=SA) \| \|  \| 24 \| randomized.ab. \| 442267 \| Advanced \| [Display Results](http://ovidsp.tx.ovid.com/sp-3.27.1a/ovidweb.cgi?&S=DPDPFPJMBHDDHOGKNCFKKEIBELHNAA00&SELECT=S.sh%7c&R=24&Process+Action=display)  [More](http://ovidsp.tx.ovid.com/sp-3.27.1a/ovidweb.cgi?&S=DPDPFPJMBHDDHOGKNCFKKEIBELHNAA00&SELECT=S.sh%7c&Expand=1&Main+Search+Page=Main+Search+Page) \| [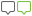](http://ovidsp.tx.ovid.com/sp-3.27.1a/ovidweb.cgi?&S=DPDPFPJMBHDDHOGKNCFKKEIBELHNAA00&R=24&Search+Annotations+Options=SA) \| \|  \| 25 \| placebo.ab. \| 205474 \| Advanced \| [Display Results](http://ovidsp.tx.ovid.com/sp-3.27.1a/ovidweb.cgi?&S=DPDPFPJMBHDDHOGKNCFKKEIBELHNAA00&SELECT=S.sh%7c&R=25&Process+Action=display)  [More](http://ovidsp.tx.ovid.com/sp-3.27.1a/ovidweb.cgi?&S=DPDPFPJMBHDDHOGKNCFKKEIBELHNAA00&SELECT=S.sh%7c&Expand=1&Main+Search+Page=Main+Search+Page) \| [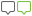](http://ovidsp.tx.ovid.com/sp-3.27.1a/ovidweb.cgi?&S=DPDPFPJMBHDDHOGKNCFKKEIBELHNAA00&R=25&Search+Annotations+Options=SA) \| \|  \| 26 \| drug therapy.fs. \| 2147127 \| Advanced \| [Display Results](http://ovidsp.tx.ovid.com/sp-3.27.1a/ovidweb.cgi?&S=DPDPFPJMBHDDHOGKNCFKKEIBELHNAA00&SELECT=S.sh%7c&R=26&Process+Action=display)  [More](http://ovidsp.tx.ovid.com/sp-3.27.1a/ovidweb.cgi?&S=DPDPFPJMBHDDHOGKNCFKKEIBELHNAA00&SELECT=S.sh%7c&Expand=1&Main+Search+Page=Main+Search+Page) \| [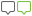](http://ovidsp.tx.ovid.com/sp-3.27.1a/ovidweb.cgi?&S=DPDPFPJMBHDDHOGKNCFKKEIBELHNAA00&R=26&Search+Annotations+Options=SA) \| \|  \| 27 \| randomly.ab. \| 305249 \| Advanced \| [Display Results](http://ovidsp.tx.ovid.com/sp-3.27.1a/ovidweb.cgi?&S=DPDPFPJMBHDDHOGKNCFKKEIBELHNAA00&SELECT=S.sh%7c&R=27&Process+Action=display)  [More](http://ovidsp.tx.ovid.com/sp-3.27.1a/ovidweb.cgi?&S=DPDPFPJMBHDDHOGKNCFKKEIBELHNAA00&SELECT=S.sh%7c&Expand=1&Main+Search+Page=Main+Search+Page) \| [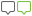](http://ovidsp.tx.ovid.com/sp-3.27.1a/ovidweb.cgi?&S=DPDPFPJMBHDDHOGKNCFKKEIBELHNAA00&R=27&Search+Annotations+Options=SA) \| \|  \| 28 \| trial.ab. \| 465908 \| Advanced \| [Display Results](http://ovidsp.tx.ovid.com/sp-3.27.1a/ovidweb.cgi?&S=DPDPFPJMBHDDHOGKNCFKKEIBELHNAA00&SELECT=S.sh%7c&R=28&Process+Action=display)  [More](http://ovidsp.tx.ovid.com/sp-3.27.1a/ovidweb.cgi?&S=DPDPFPJMBHDDHOGKNCFKKEIBELHNAA00&SELECT=S.sh%7c&Expand=1&Main+Search+Page=Main+Search+Page) \| [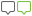](http://ovidsp.tx.ovid.com/sp-3.27.1a/ovidweb.cgi?&S=DPDPFPJMBHDDHOGKNCFKKEIBELHNAA00&R=28&Search+Annotations+Options=SA) \| \|  \| 29 \| groups.ab. \| 1885345 \| Advanced \| [Display Results](http://ovidsp.tx.ovid.com/sp-3.27.1a/ovidweb.cgi?&S=DPDPFPJMBHDDHOGKNCFKKEIBELHNAA00&SELECT=S.sh%7c&R=29&Process+Action=display)  [More](http://ovidsp.tx.ovid.com/sp-3.27.1a/ovidweb.cgi?&S=DPDPFPJMBHDDHOGKNCFKKEIBELHNAA00&SELECT=S.sh%7c&Expand=1&Main+Search+Page=Main+Search+Page) \| [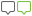](http://ovidsp.tx.ovid.com/sp-3.27.1a/ovidweb.cgi?&S=DPDPFPJMBHDDHOGKNCFKKEIBELHNAA00&R=29&Search+Annotations+Options=SA) \| \|  \| 30 \| 22 or 23 or 24 or 25 or 26 or 27 or 28 or 29 \| 4448873 \| Advanced \| [Display Results](http://ovidsp.tx.ovid.com/sp-3.27.1a/ovidweb.cgi?&S=DPDPFPJMBHDDHOGKNCFKKEIBELHNAA00&SELECT=S.sh%7c&R=30&Process+Action=display)  [More](http://ovidsp.tx.ovid.com/sp-3.27.1a/ovidweb.cgi?&S=DPDPFPJMBHDDHOGKNCFKKEIBELHNAA00&SELECT=S.sh%7c&Expand=1&Main+Search+Page=Main+Search+Page) \| [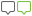](http://ovidsp.tx.ovid.com/sp-3.27.1a/ovidweb.cgi?&S=DPDPFPJMBHDDHOGKNCFKKEIBELHNAA00&R=30&Search+Annotations+Options=SA) \| \|  \| 31 \| 21 and 30 \| 520 \| Advanced \| [Display Results](http://ovidsp.tx.ovid.com/sp-3.27.1a/ovidweb.cgi?&S=DPDPFPJMBHDDHOGKNCFKKEIBELHNAA00&SELECT=S.sh%7c&R=31&Process+Action=display)  [More](http://ovidsp.tx.ovid.com/sp-3.27.1a/ovidweb.cgi?&S=DPDPFPJMBHDDHOGKNCFKKEIBELHNAA00&SELECT=S.sh%7c&Expand=1&Main+Search+Page=Main+Search+Page) \| [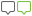](http://ovidsp.tx.ovid.com/sp-3.27.1a/ovidweb.cgi?&S=DPDPFPJMBHDDHOGKNCFKKEIBELHNAA00&R=31&Search+Annotations+Options=SA) \| \|  \| \| \|  \|  \|  \|  \|   **EMBASE: (24/11/17)**   \|  \|  \| \| --- \| --- \| \|  \|  \|  \|  \| \| --- \| --- \| --- \| --- \| --- \| --- \| --- \| --- \| --- \| --- \| --- \| --- \| --- \| --- \| --- \| --- \| --- \| --- \| --- \| --- \| --- \| --- \| --- \| --- \| --- \| --- \| --- \| --- \| --- \| --- \| --- \| --- \| --- \| --- \| --- \| --- \| --- \| --- \| --- \| --- \| --- \| --- \| --- \| --- \| --- \| --- \| --- \| --- \| --- \| --- \| --- \| --- \| --- \| --- \| --- \| --- \| --- \| --- \| --- \| --- \| --- \| --- \| --- \| --- \| --- \| --- \| --- \| --- \| --- \| --- \| --- \| --- \| --- \| --- \| --- \| --- \| --- \| --- \| --- \| --- \| --- \| --- \| --- \| --- \| --- \| --- \| --- \| --- \| --- \| --- \| --- \| --- \| --- \| --- \| --- \| --- \| --- \| --- \| --- \| --- \| --- \| --- \| --- \| --- \| --- \| --- \| --- \| --- \| --- \| --- \| --- \| --- \| --- \| --- \| --- \| --- \| --- \| --- \| --- \| --- \| --- \| --- \| --- \| --- \| --- \| --- \| --- \| --- \| --- \| --- \| --- \| --- \| --- \| --- \| --- \| --- \| --- \| --- \| --- \| --- \| --- \| --- \| --- \| --- \| --- \| --- \| --- \| --- \| --- \| --- \| --- \| --- \| --- \| --- \| --- \| --- \| --- \| --- \| --- \| --- \| --- \| --- \| --- \| --- \| --- \| --- \| --- \| --- \| --- \| --- \| --- \| --- \| --- \| --- \| --- \| --- \| --- \| --- \| --- \| --- \| --- \| --- \| --- \| --- \| --- \| --- \| --- \| --- \| --- \| --- \| --- \| --- \| --- \| --- \| --- \| --- \| --- \| --- \| --- \| --- \| --- \| --- \| --- \| --- \| --- \| --- \| --- \| --- \| --- \| --- \| --- \| --- \| --- \| --- \| --- \| --- \| --- \| --- \| --- \| --- \| --- \| --- \| --- \| --- \| --- \| --- \| --- \| --- \| --- \| --- \| --- \| --- \| --- \| --- \| --- \| --- \| --- \| --- \| --- \| --- \| --- \| --- \| --- \| \| \| **Searches** \| **Results** \| **Type** \| **Actions** \| **Annotations** \| \| --- \| --- \| --- \| --- \| --- \| \|  \| \| \| \| \| \| \| \|  \| 1 \| *patellar ligament/ \| 206 \| Advanced \| [Display Results](http://ovidsp.tx.ovid.com/sp-3.27.1a/ovidweb.cgi?&S=DPDPFPJMBHDDHOGKNCFKKEIBELHNAA00&SELECT=S.sh%7c&R=1&Process+Action=display)  [More](http://ovidsp.tx.ovid.com/sp-3.27.1a/ovidweb.cgi?&S=DPDPFPJMBHDDHOGKNCFKKEIBELHNAA00&SELECT=S.sh%7c&Expand=1&Main+Search+Page=Main+Search+Page) \| [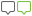](http://ovidsp.tx.ovid.com/sp-3.27.1a/ovidweb.cgi?&S=DPDPFPJMBHDDHOGKNCFKKEIBELHNAA00&R=1&Search+Annotations+Options=SA) \| \|  \| 2 \| patellar ligament/ \| 687 \| Advanced \| [Display Results](http://ovidsp.tx.ovid.com/sp-3.27.1a/ovidweb.cgi?&S=DPDPFPJMBHDDHOGKNCFKKEIBELHNAA00&SELECT=S.sh%7c&R=2&Process+Action=display)  [More](http://ovidsp.tx.ovid.com/sp-3.27.1a/ovidweb.cgi?&S=DPDPFPJMBHDDHOGKNCFKKEIBELHNAA00&SELECT=S.sh%7c&Expand=1&Main+Search+Page=Main+Search+Page) \| [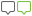](http://ovidsp.tx.ovid.com/sp-3.27.1a/ovidweb.cgi?&S=DPDPFPJMBHDDHOGKNCFKKEIBELHNAA00&R=2&Search+Annotations+Options=SA) \| \|  \| 3 \| *patella/ \| 3675 \| Advanced \| [Display Results](http://ovidsp.tx.ovid.com/sp-3.27.1a/ovidweb.cgi?&S=DPDPFPJMBHDDHOGKNCFKKEIBELHNAA00&SELECT=S.sh%7c&R=3&Process+Action=display)  [More](http://ovidsp.tx.ovid.com/sp-3.27.1a/ovidweb.cgi?&S=DPDPFPJMBHDDHOGKNCFKKEIBELHNAA00&SELECT=S.sh%7c&Expand=1&Main+Search+Page=Main+Search+Page) \| [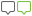](http://ovidsp.tx.ovid.com/sp-3.27.1a/ovidweb.cgi?&S=DPDPFPJMBHDDHOGKNCFKKEIBELHNAA00&R=3&Search+Annotations+Options=SA) \| \|  \| 4 \| patella/ \| 8700 \| Advanced \| [Display Results](http://ovidsp.tx.ovid.com/sp-3.27.1a/ovidweb.cgi?&S=DPDPFPJMBHDDHOGKNCFKKEIBELHNAA00&SELECT=S.sh%7c&R=4&Process+Action=display)  [More](http://ovidsp.tx.ovid.com/sp-3.27.1a/ovidweb.cgi?&S=DPDPFPJMBHDDHOGKNCFKKEIBELHNAA00&SELECT=S.sh%7c&Expand=1&Main+Search+Page=Main+Search+Page) \| [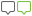](http://ovidsp.tx.ovid.com/sp-3.27.1a/ovidweb.cgi?&S=DPDPFPJMBHDDHOGKNCFKKEIBELHNAA00&R=4&Search+Annotations+Options=SA) \| \|  \| 5 \| exp *tendinitis/ \| 6557 \| Advanced \| [Display Results](http://ovidsp.tx.ovid.com/sp-3.27.1a/ovidweb.cgi?&S=DPDPFPJMBHDDHOGKNCFKKEIBELHNAA00&SELECT=S.sh%7c&R=5&Process+Action=display)  [More](http://ovidsp.tx.ovid.com/sp-3.27.1a/ovidweb.cgi?&S=DPDPFPJMBHDDHOGKNCFKKEIBELHNAA00&SELECT=S.sh%7c&Expand=1&Main+Search+Page=Main+Search+Page) \| [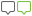](http://ovidsp.tx.ovid.com/sp-3.27.1a/ovidweb.cgi?&S=DPDPFPJMBHDDHOGKNCFKKEIBELHNAA00&R=5&Search+Annotations+Options=SA) \| \|  \| 6 \| exp tendinitis/ \| 14755 \| Advanced \| [Display Results](http://ovidsp.tx.ovid.com/sp-3.27.1a/ovidweb.cgi?&S=DPDPFPJMBHDDHOGKNCFKKEIBELHNAA00&SELECT=S.sh%7c&R=6&Process+Action=display)  [More](http://ovidsp.tx.ovid.com/sp-3.27.1a/ovidweb.cgi?&S=DPDPFPJMBHDDHOGKNCFKKEIBELHNAA00&SELECT=S.sh%7c&Expand=1&Main+Search+Page=Main+Search+Page) \| [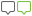](http://ovidsp.tx.ovid.com/sp-3.27.1a/ovidweb.cgi?&S=DPDPFPJMBHDDHOGKNCFKKEIBELHNAA00&R=6&Search+Annotations+Options=SA) \| \|  \| 7 \| 3 and 5 \| 47 \| Advanced \| [Display Results](http://ovidsp.tx.ovid.com/sp-3.27.1a/ovidweb.cgi?&S=DPDPFPJMBHDDHOGKNCFKKEIBELHNAA00&SELECT=S.sh%7c&R=7&Process+Action=display)  [More](http://ovidsp.tx.ovid.com/sp-3.27.1a/ovidweb.cgi?&S=DPDPFPJMBHDDHOGKNCFKKEIBELHNAA00&SELECT=S.sh%7c&Expand=1&Main+Search+Page=Main+Search+Page) \| [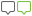](http://ovidsp.tx.ovid.com/sp-3.27.1a/ovidweb.cgi?&S=DPDPFPJMBHDDHOGKNCFKKEIBELHNAA00&R=7&Search+Annotations+Options=SA) \| \|  \| 8 \| 4 and 6 \| 140 \| Advanced \| [Display Results](http://ovidsp.tx.ovid.com/sp-3.27.1a/ovidweb.cgi?&S=DPDPFPJMBHDDHOGKNCFKKEIBELHNAA00&SELECT=S.sh%7c&R=8&Process+Action=display)  [More](http://ovidsp.tx.ovid.com/sp-3.27.1a/ovidweb.cgi?&S=DPDPFPJMBHDDHOGKNCFKKEIBELHNAA00&SELECT=S.sh%7c&Expand=1&Main+Search+Page=Main+Search+Page) \| [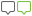](http://ovidsp.tx.ovid.com/sp-3.27.1a/ovidweb.cgi?&S=DPDPFPJMBHDDHOGKNCFKKEIBELHNAA00&R=8&Search+Annotations+Options=SA) \| \|  \| 9 \| exp *tendon injury/ \| 12698 \| Advanced \| [Display Results](http://ovidsp.tx.ovid.com/sp-3.27.1a/ovidweb.cgi?&S=DPDPFPJMBHDDHOGKNCFKKEIBELHNAA00&SELECT=S.sh%7c&R=9&Process+Action=display)  [More](http://ovidsp.tx.ovid.com/sp-3.27.1a/ovidweb.cgi?&S=DPDPFPJMBHDDHOGKNCFKKEIBELHNAA00&SELECT=S.sh%7c&Expand=1&Main+Search+Page=Main+Search+Page) \| [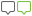](http://ovidsp.tx.ovid.com/sp-3.27.1a/ovidweb.cgi?&S=DPDPFPJMBHDDHOGKNCFKKEIBELHNAA00&R=9&Search+Annotations+Options=SA) \| \|  \| 10 \| exp tendon injury/ \| 20079 \| Advanced \| [Display Results](http://ovidsp.tx.ovid.com/sp-3.27.1a/ovidweb.cgi?&S=DPDPFPJMBHDDHOGKNCFKKEIBELHNAA00&SELECT=S.sh%7c&R=10&Process+Action=display)  [More](http://ovidsp.tx.ovid.com/sp-3.27.1a/ovidweb.cgi?&S=DPDPFPJMBHDDHOGKNCFKKEIBELHNAA00&SELECT=S.sh%7c&Expand=1&Main+Search+Page=Main+Search+Page) \| [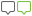](http://ovidsp.tx.ovid.com/sp-3.27.1a/ovidweb.cgi?&S=DPDPFPJMBHDDHOGKNCFKKEIBELHNAA00&R=10&Search+Annotations+Options=SA) \| \|  \| 11 \| 3 and 9 \| 65 \| Advanced \| [Display Results](http://ovidsp.tx.ovid.com/sp-3.27.1a/ovidweb.cgi?&S=DPDPFPJMBHDDHOGKNCFKKEIBELHNAA00&SELECT=S.sh%7c&R=11&Process+Action=display)  [More](http://ovidsp.tx.ovid.com/sp-3.27.1a/ovidweb.cgi?&S=DPDPFPJMBHDDHOGKNCFKKEIBELHNAA00&SELECT=S.sh%7c&Expand=1&Main+Search+Page=Main+Search+Page) \| [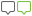](http://ovidsp.tx.ovid.com/sp-3.27.1a/ovidweb.cgi?&S=DPDPFPJMBHDDHOGKNCFKKEIBELHNAA00&R=11&Search+Annotations+Options=SA) \| \|  \| 12 \| 4 and 10 \| 206 \| Advanced \| [Display Results](http://ovidsp.tx.ovid.com/sp-3.27.1a/ovidweb.cgi?&S=DPDPFPJMBHDDHOGKNCFKKEIBELHNAA00&SELECT=S.sh%7c&R=12&Process+Action=display)  [More](http://ovidsp.tx.ovid.com/sp-3.27.1a/ovidweb.cgi?&S=DPDPFPJMBHDDHOGKNCFKKEIBELHNAA00&SELECT=S.sh%7c&Expand=1&Main+Search+Page=Main+Search+Page) \| [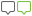](http://ovidsp.tx.ovid.com/sp-3.27.1a/ovidweb.cgi?&S=DPDPFPJMBHDDHOGKNCFKKEIBELHNAA00&R=12&Search+Annotations+Options=SA) \| \|  \| 13 \| ((patella* or kneecap* or knee cap*) adj3 (tendon* or tendin*)).ti,ab. \| 6355 \| Advanced \| [Display Results](http://ovidsp.tx.ovid.com/sp-3.27.1a/ovidweb.cgi?&S=DPDPFPJMBHDDHOGKNCFKKEIBELHNAA00&SELECT=S.sh%7c&R=13&Process+Action=display)  [More](http://ovidsp.tx.ovid.com/sp-3.27.1a/ovidweb.cgi?&S=DPDPFPJMBHDDHOGKNCFKKEIBELHNAA00&SELECT=S.sh%7c&Expand=1&Main+Search+Page=Main+Search+Page) \| [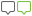](http://ovidsp.tx.ovid.com/sp-3.27.1a/ovidweb.cgi?&S=DPDPFPJMBHDDHOGKNCFKKEIBELHNAA00&R=13&Search+Annotations+Options=SA) \| \|  \| 14 \| osgood schlatter*.ti,ab. \| 364 \| Advanced \| [Display Results](http://ovidsp.tx.ovid.com/sp-3.27.1a/ovidweb.cgi?&S=DPDPFPJMBHDDHOGKNCFKKEIBELHNAA00&SELECT=S.sh%7c&R=14&Process+Action=display)  [More](http://ovidsp.tx.ovid.com/sp-3.27.1a/ovidweb.cgi?&S=DPDPFPJMBHDDHOGKNCFKKEIBELHNAA00&SELECT=S.sh%7c&Expand=1&Main+Search+Page=Main+Search+Page) \| [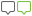](http://ovidsp.tx.ovid.com/sp-3.27.1a/ovidweb.cgi?&S=DPDPFPJMBHDDHOGKNCFKKEIBELHNAA00&R=14&Search+Annotations+Options=SA) \| \|  \| 15 \| sinding larsen johansson*.ti,ab. \| 51 \| Advanced \| [Display Results](http://ovidsp.tx.ovid.com/sp-3.27.1a/ovidweb.cgi?&S=DPDPFPJMBHDDHOGKNCFKKEIBELHNAA00&SELECT=S.sh%7c&R=15&Process+Action=display)  [More](http://ovidsp.tx.ovid.com/sp-3.27.1a/ovidweb.cgi?&S=DPDPFPJMBHDDHOGKNCFKKEIBELHNAA00&SELECT=S.sh%7c&Expand=1&Main+Search+Page=Main+Search+Page) \| [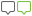](http://ovidsp.tx.ovid.com/sp-3.27.1a/ovidweb.cgi?&S=DPDPFPJMBHDDHOGKNCFKKEIBELHNAA00&R=15&Search+Annotations+Options=SA) \| \|  \| 16 \| jumpers knee*.ti,ab. \| 229 \| Advanced \| [Display Results](http://ovidsp.tx.ovid.com/sp-3.27.1a/ovidweb.cgi?&S=DPDPFPJMBHDDHOGKNCFKKEIBELHNAA00&SELECT=S.sh%7c&R=16&Process+Action=display)  [More](http://ovidsp.tx.ovid.com/sp-3.27.1a/ovidweb.cgi?&S=DPDPFPJMBHDDHOGKNCFKKEIBELHNAA00&SELECT=S.sh%7c&Expand=1&Main+Search+Page=Main+Search+Page) \| [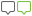](http://ovidsp.tx.ovid.com/sp-3.27.1a/ovidweb.cgi?&S=DPDPFPJMBHDDHOGKNCFKKEIBELHNAA00&R=16&Search+Annotations+Options=SA) \| \|  \| 17 \| (apophy* adj3 tibia* tuber*).ti,ab. \| 30 \| Advanced \| [Display Results](http://ovidsp.tx.ovid.com/sp-3.27.1a/ovidweb.cgi?&S=DPDPFPJMBHDDHOGKNCFKKEIBELHNAA00&SELECT=S.sh%7c&R=17&Process+Action=display)  [More](http://ovidsp.tx.ovid.com/sp-3.27.1a/ovidweb.cgi?&S=DPDPFPJMBHDDHOGKNCFKKEIBELHNAA00&SELECT=S.sh%7c&Expand=1&Main+Search+Page=Main+Search+Page) \| [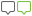](http://ovidsp.tx.ovid.com/sp-3.27.1a/ovidweb.cgi?&S=DPDPFPJMBHDDHOGKNCFKKEIBELHNAA00&R=17&Search+Annotations+Options=SA) \| \|  \| 18 \| lannelongue*.ti,ab. \| 69 \| Advanced \| [Display Results](http://ovidsp.tx.ovid.com/sp-3.27.1a/ovidweb.cgi?&S=DPDPFPJMBHDDHOGKNCFKKEIBELHNAA00&SELECT=S.sh%7c&R=18&Process+Action=display)  [More](http://ovidsp.tx.ovid.com/sp-3.27.1a/ovidweb.cgi?&S=DPDPFPJMBHDDHOGKNCFKKEIBELHNAA00&SELECT=S.sh%7c&Expand=1&Main+Search+Page=Main+Search+Page) \| [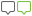](http://ovidsp.tx.ovid.com/sp-3.27.1a/ovidweb.cgi?&S=DPDPFPJMBHDDHOGKNCFKKEIBELHNAA00&R=18&Search+Annotations+Options=SA) \| \|  \| 19 \| (lannelongue* adj3 (disease* or syndrom* or condition* or disorder*)).ti,ab. \| 1 \| Advanced \| [Display Results](http://ovidsp.tx.ovid.com/sp-3.27.1a/ovidweb.cgi?&S=DPDPFPJMBHDDHOGKNCFKKEIBELHNAA00&SELECT=S.sh%7c&R=19&Process+Action=display)  [More](http://ovidsp.tx.ovid.com/sp-3.27.1a/ovidweb.cgi?&S=DPDPFPJMBHDDHOGKNCFKKEIBELHNAA00&SELECT=S.sh%7c&Expand=1&Main+Search+Page=Main+Search+Page) \| [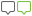](http://ovidsp.tx.ovid.com/sp-3.27.1a/ovidweb.cgi?&S=DPDPFPJMBHDDHOGKNCFKKEIBELHNAA00&R=19&Search+Annotations+Options=SA) \| \|  \| 20 \| 7 or 11 or 13 or 14 or 15 or 16 or 17 or 18 \| 6837 \| Advanced \| [Display Results](http://ovidsp.tx.ovid.com/sp-3.27.1a/ovidweb.cgi?&S=DPDPFPJMBHDDHOGKNCFKKEIBELHNAA00&SELECT=S.sh%7c&R=20&Process+Action=display)  [More](http://ovidsp.tx.ovid.com/sp-3.27.1a/ovidweb.cgi?&S=DPDPFPJMBHDDHOGKNCFKKEIBELHNAA00&SELECT=S.sh%7c&Expand=1&Main+Search+Page=Main+Search+Page) \| [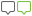](http://ovidsp.tx.ovid.com/sp-3.27.1a/ovidweb.cgi?&S=DPDPFPJMBHDDHOGKNCFKKEIBELHNAA00&R=20&Search+Annotations+Options=SA) \| \|  \| 21 \| 8 or 12 or 13 or 14 or 15 or 16 or 17 or 18 \| 6951 \| Advanced \| [Display Results](http://ovidsp.tx.ovid.com/sp-3.27.1a/ovidweb.cgi?&S=DPDPFPJMBHDDHOGKNCFKKEIBELHNAA00&SELECT=S.sh%7c&R=21&Process+Action=display)  [More](http://ovidsp.tx.ovid.com/sp-3.27.1a/ovidweb.cgi?&S=DPDPFPJMBHDDHOGKNCFKKEIBELHNAA00&SELECT=S.sh%7c&Expand=1&Main+Search+Page=Main+Search+Page) \| [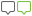](http://ovidsp.tx.ovid.com/sp-3.27.1a/ovidweb.cgi?&S=DPDPFPJMBHDDHOGKNCFKKEIBELHNAA00&R=21&Search+Annotations+Options=SA) \| \|  \| 22 \| exp *pediatrics/ \| 53088 \| Advanced \| [Display Results](http://ovidsp.tx.ovid.com/sp-3.27.1a/ovidweb.cgi?&S=DPDPFPJMBHDDHOGKNCFKKEIBELHNAA00&SELECT=S.sh%7c&R=22&Process+Action=display)  [More](http://ovidsp.tx.ovid.com/sp-3.27.1a/ovidweb.cgi?&S=DPDPFPJMBHDDHOGKNCFKKEIBELHNAA00&SELECT=S.sh%7c&Expand=1&Main+Search+Page=Main+Search+Page) \| [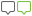](http://ovidsp.tx.ovid.com/sp-3.27.1a/ovidweb.cgi?&S=DPDPFPJMBHDDHOGKNCFKKEIBELHNAA00&R=22&Search+Annotations+Options=SA) \| \|  \| 23 \| exp pediatrics/ \| 96414 \| Advanced \| [Display Results](http://ovidsp.tx.ovid.com/sp-3.27.1a/ovidweb.cgi?&S=DPDPFPJMBHDDHOGKNCFKKEIBELHNAA00&SELECT=S.sh%7c&R=23&Process+Action=display)  [More](http://ovidsp.tx.ovid.com/sp-3.27.1a/ovidweb.cgi?&S=DPDPFPJMBHDDHOGKNCFKKEIBELHNAA00&SELECT=S.sh%7c&Expand=1&Main+Search+Page=Main+Search+Page) \| [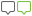](http://ovidsp.tx.ovid.com/sp-3.27.1a/ovidweb.cgi?&S=DPDPFPJMBHDDHOGKNCFKKEIBELHNAA00&R=23&Search+Annotations+Options=SA) \| \|  \| 24 \| exp *Child/ \| 166770 \| Advanced \| [Display Results](http://ovidsp.tx.ovid.com/sp-3.27.1a/ovidweb.cgi?&S=DPDPFPJMBHDDHOGKNCFKKEIBELHNAA00&SELECT=S.sh%7c&R=24&Process+Action=display)  [More](http://ovidsp.tx.ovid.com/sp-3.27.1a/ovidweb.cgi?&S=DPDPFPJMBHDDHOGKNCFKKEIBELHNAA00&SELECT=S.sh%7c&Expand=1&Main+Search+Page=Main+Search+Page) \| [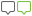](http://ovidsp.tx.ovid.com/sp-3.27.1a/ovidweb.cgi?&S=DPDPFPJMBHDDHOGKNCFKKEIBELHNAA00&R=24&Search+Annotations+Options=SA) \| \|  \| 25 \| exp Child/ \| 2492089 \| Advanced \| [Display Results](http://ovidsp.tx.ovid.com/sp-3.27.1a/ovidweb.cgi?&S=DPDPFPJMBHDDHOGKNCFKKEIBELHNAA00&SELECT=S.sh%7c&R=25&Process+Action=display)  [More](http://ovidsp.tx.ovid.com/sp-3.27.1a/ovidweb.cgi?&S=DPDPFPJMBHDDHOGKNCFKKEIBELHNAA00&SELECT=S.sh%7c&Expand=1&Main+Search+Page=Main+Search+Page) \| [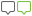](http://ovidsp.tx.ovid.com/sp-3.27.1a/ovidweb.cgi?&S=DPDPFPJMBHDDHOGKNCFKKEIBELHNAA00&R=25&Search+Annotations+Options=SA) \| \|  \| 26 \| exp *Infant/ \| 62245 \| Advanced \| [Display Results](http://ovidsp.tx.ovid.com/sp-3.27.1a/ovidweb.cgi?&S=DPDPFPJMBHDDHOGKNCFKKEIBELHNAA00&SELECT=S.sh%7c&R=26&Process+Action=display)  [More](http://ovidsp.tx.ovid.com/sp-3.27.1a/ovidweb.cgi?&S=DPDPFPJMBHDDHOGKNCFKKEIBELHNAA00&SELECT=S.sh%7c&Expand=1&Main+Search+Page=Main+Search+Page) \| [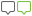](http://ovidsp.tx.ovid.com/sp-3.27.1a/ovidweb.cgi?&S=DPDPFPJMBHDDHOGKNCFKKEIBELHNAA00&R=26&Search+Annotations+Options=SA) \| \|  \| 27 \| exp Infant/ \| 982585 \| Advanced \| [Display Results](http://ovidsp.tx.ovid.com/sp-3.27.1a/ovidweb.cgi?&S=DPDPFPJMBHDDHOGKNCFKKEIBELHNAA00&SELECT=S.sh%7c&R=27&Process+Action=display)  [More](http://ovidsp.tx.ovid.com/sp-3.27.1a/ovidweb.cgi?&S=DPDPFPJMBHDDHOGKNCFKKEIBELHNAA00&SELECT=S.sh%7c&Expand=1&Main+Search+Page=Main+Search+Page) \| [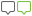](http://ovidsp.tx.ovid.com/sp-3.27.1a/ovidweb.cgi?&S=DPDPFPJMBHDDHOGKNCFKKEIBELHNAA00&R=27&Search+Annotations+Options=SA) \| \|  \| 28 \| exp *Adolescent/ \| 29459 \| Advanced \| [Display Results](http://ovidsp.tx.ovid.com/sp-3.27.1a/ovidweb.cgi?&S=DPDPFPJMBHDDHOGKNCFKKEIBELHNAA00&SELECT=S.sh%7c&R=28&Process+Action=display)  [More](http://ovidsp.tx.ovid.com/sp-3.27.1a/ovidweb.cgi?&S=DPDPFPJMBHDDHOGKNCFKKEIBELHNAA00&SELECT=S.sh%7c&Expand=1&Main+Search+Page=Main+Search+Page) \| [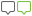](http://ovidsp.tx.ovid.com/sp-3.27.1a/ovidweb.cgi?&S=DPDPFPJMBHDDHOGKNCFKKEIBELHNAA00&R=28&Search+Annotations+Options=SA) \| \|  \| 29 \| exp Adolescent/ \| 1463129 \| Advanced \| [Display Results](http://ovidsp.tx.ovid.com/sp-3.27.1a/ovidweb.cgi?&S=DPDPFPJMBHDDHOGKNCFKKEIBELHNAA00&SELECT=S.sh%7c&R=29&Process+Action=display)  [More](http://ovidsp.tx.ovid.com/sp-3.27.1a/ovidweb.cgi?&S=DPDPFPJMBHDDHOGKNCFKKEIBELHNAA00&SELECT=S.sh%7c&Expand=1&Main+Search+Page=Main+Search+Page) \| [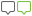](http://ovidsp.tx.ovid.com/sp-3.27.1a/ovidweb.cgi?&S=DPDPFPJMBHDDHOGKNCFKKEIBELHNAA00&R=29&Search+Annotations+Options=SA) \| \|  \| 30 \| (child* or infant* or paediatric* or pediatric* or adolescen* or teen* or youth* or boy* or girl* neonat* or baby or babies or babys).ti,ab. \| 2282313 \| Advanced \| [Display Results](http://ovidsp.tx.ovid.com/sp-3.27.1a/ovidweb.cgi?&S=DPDPFPJMBHDDHOGKNCFKKEIBELHNAA00&SELECT=S.sh%7c&R=30&Process+Action=display)  [More](http://ovidsp.tx.ovid.com/sp-3.27.1a/ovidweb.cgi?&S=DPDPFPJMBHDDHOGKNCFKKEIBELHNAA00&SELECT=S.sh%7c&Expand=1&Main+Search+Page=Main+Search+Page) \| [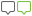](http://ovidsp.tx.ovid.com/sp-3.27.1a/ovidweb.cgi?&S=DPDPFPJMBHDDHOGKNCFKKEIBELHNAA00&R=30&Search+Annotations+Options=SA) \| \|  \| 31 \| 22 or 24 or 26 or 28 or 30 \| 2332891 \| Advanced \| [Display Results](http://ovidsp.tx.ovid.com/sp-3.27.1a/ovidweb.cgi?&S=DPDPFPJMBHDDHOGKNCFKKEIBELHNAA00&SELECT=S.sh%7c&R=31&Process+Action=display)  [More](http://ovidsp.tx.ovid.com/sp-3.27.1a/ovidweb.cgi?&S=DPDPFPJMBHDDHOGKNCFKKEIBELHNAA00&SELECT=S.sh%7c&Expand=1&Main+Search+Page=Main+Search+Page) \| [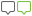](http://ovidsp.tx.ovid.com/sp-3.27.1a/ovidweb.cgi?&S=DPDPFPJMBHDDHOGKNCFKKEIBELHNAA00&R=31&Search+Annotations+Options=SA) \| \|  \| 32 \| 23 or 25 or 27 or 29 or 30 \| 3860088 \| Advanced \| [Display Results](http://ovidsp.tx.ovid.com/sp-3.27.1a/ovidweb.cgi?&S=DPDPFPJMBHDDHOGKNCFKKEIBELHNAA00&SELECT=S.sh%7c&R=32&Process+Action=display)  [More](http://ovidsp.tx.ovid.com/sp-3.27.1a/ovidweb.cgi?&S=DPDPFPJMBHDDHOGKNCFKKEIBELHNAA00&SELECT=S.sh%7c&Expand=1&Main+Search+Page=Main+Search+Page) \| [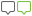](http://ovidsp.tx.ovid.com/sp-3.27.1a/ovidweb.cgi?&S=DPDPFPJMBHDDHOGKNCFKKEIBELHNAA00&R=32&Search+Annotations+Options=SA) \| \|  \| 33 \| 20 and 31 \| 443 \| Advanced \| [Display Results](http://ovidsp.tx.ovid.com/sp-3.27.1a/ovidweb.cgi?&S=DPDPFPJMBHDDHOGKNCFKKEIBELHNAA00&SELECT=S.sh%7c&R=33&Process+Action=display)  [More](http://ovidsp.tx.ovid.com/sp-3.27.1a/ovidweb.cgi?&S=DPDPFPJMBHDDHOGKNCFKKEIBELHNAA00&SELECT=S.sh%7c&Expand=1&Main+Search+Page=Main+Search+Page) \| [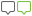](http://ovidsp.tx.ovid.com/sp-3.27.1a/ovidweb.cgi?&S=DPDPFPJMBHDDHOGKNCFKKEIBELHNAA00&R=33&Search+Annotations+Options=SA) \| \|  \| 34 \| 21 and 32 \| 1427 \| Advanced \| [Display Results](http://ovidsp.tx.ovid.com/sp-3.27.1a/ovidweb.cgi?&S=DPDPFPJMBHDDHOGKNCFKKEIBELHNAA00&SELECT=S.sh%7c&R=34&Process+Action=display)  [More](http://ovidsp.tx.ovid.com/sp-3.27.1a/ovidweb.cgi?&S=DPDPFPJMBHDDHOGKNCFKKEIBELHNAA00&SELECT=S.sh%7c&Expand=1&Main+Search+Page=Main+Search+Page) \| [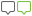](http://ovidsp.tx.ovid.com/sp-3.27.1a/ovidweb.cgi?&S=DPDPFPJMBHDDHOGKNCFKKEIBELHNAA00&R=34&Search+Annotations+Options=SA) \| \|  \| 35 \| randomized controlled trial/ \| 483842 \| Advanced \| [Display Results](http://ovidsp.tx.ovid.com/sp-3.27.1a/ovidweb.cgi?&S=DPDPFPJMBHDDHOGKNCFKKEIBELHNAA00&SELECT=S.sh%7c&R=35&Process+Action=display)  [More](http://ovidsp.tx.ovid.com/sp-3.27.1a/ovidweb.cgi?&S=DPDPFPJMBHDDHOGKNCFKKEIBELHNAA00&SELECT=S.sh%7c&Expand=1&Main+Search+Page=Main+Search+Page) \| [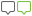](http://ovidsp.tx.ovid.com/sp-3.27.1a/ovidweb.cgi?&S=DPDPFPJMBHDDHOGKNCFKKEIBELHNAA00&R=35&Search+Annotations+Options=SA) \| \|  \| 36 \| single blind procedure/ or double blind procedure/ \| 174315 \| Advanced \| [Display Results](http://ovidsp.tx.ovid.com/sp-3.27.1a/ovidweb.cgi?&S=DPDPFPJMBHDDHOGKNCFKKEIBELHNAA00&SELECT=S.sh%7c&R=36&Process+Action=display)  [More](http://ovidsp.tx.ovid.com/sp-3.27.1a/ovidweb.cgi?&S=DPDPFPJMBHDDHOGKNCFKKEIBELHNAA00&SELECT=S.sh%7c&Expand=1&Main+Search+Page=Main+Search+Page) \| [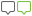](http://ovidsp.tx.ovid.com/sp-3.27.1a/ovidweb.cgi?&S=DPDPFPJMBHDDHOGKNCFKKEIBELHNAA00&R=36&Search+Annotations+Options=SA) \| \|  \| 37 \| crossover procedure/ \| 54247 \| Advanced \| [Display Results](http://ovidsp.tx.ovid.com/sp-3.27.1a/ovidweb.cgi?&S=DPDPFPJMBHDDHOGKNCFKKEIBELHNAA00&SELECT=S.sh%7c&R=37&Process+Action=display)  [More](http://ovidsp.tx.ovid.com/sp-3.27.1a/ovidweb.cgi?&S=DPDPFPJMBHDDHOGKNCFKKEIBELHNAA00&SELECT=S.sh%7c&Expand=1&Main+Search+Page=Main+Search+Page) \| [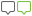](http://ovidsp.tx.ovid.com/sp-3.27.1a/ovidweb.cgi?&S=DPDPFPJMBHDDHOGKNCFKKEIBELHNAA00&R=37&Search+Annotations+Options=SA) \| \|  \| 38 \| random*.tw. \| 1265503 \| Advanced \| [Display Results](http://ovidsp.tx.ovid.com/sp-3.27.1a/ovidweb.cgi?&S=DPDPFPJMBHDDHOGKNCFKKEIBELHNAA00&SELECT=S.sh%7c&R=38&Process+Action=display)  [More](http://ovidsp.tx.ovid.com/sp-3.27.1a/ovidweb.cgi?&S=DPDPFPJMBHDDHOGKNCFKKEIBELHNAA00&SELECT=S.sh%7c&Expand=1&Main+Search+Page=Main+Search+Page) \| [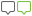](http://ovidsp.tx.ovid.com/sp-3.27.1a/ovidweb.cgi?&S=DPDPFPJMBHDDHOGKNCFKKEIBELHNAA00&R=38&Search+Annotations+Options=SA) \| \|  \| 39 \| (random or ((singl* or doubl*) adj (blind* or mask*)) or crossover or cross over or factorial* or latin square or assign* or allocat* or volunteer*).ti,ab. \| 1146486 \| Advanced \| [Display Results](http://ovidsp.tx.ovid.com/sp-3.27.1a/ovidweb.cgi?&S=DPDPFPJMBHDDHOGKNCFKKEIBELHNAA00&SELECT=S.sh%7c&R=39&Process+Action=display)  [More](http://ovidsp.tx.ovid.com/sp-3.27.1a/ovidweb.cgi?&S=DPDPFPJMBHDDHOGKNCFKKEIBELHNAA00&SELECT=S.sh%7c&Expand=1&Main+Search+Page=Main+Search+Page) \| [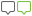](http://ovidsp.tx.ovid.com/sp-3.27.1a/ovidweb.cgi?&S=DPDPFPJMBHDDHOGKNCFKKEIBELHNAA00&R=39&Search+Annotations+Options=SA) \| \|  \| 40 \| 35 or 36 or 37 or 38 or 39 \| 1907141 \| Advanced \| [Display Results](http://ovidsp.tx.ovid.com/sp-3.27.1a/ovidweb.cgi?&S=DPDPFPJMBHDDHOGKNCFKKEIBELHNAA00&SELECT=S.sh%7c&R=40&Process+Action=display)  [More](http://ovidsp.tx.ovid.com/sp-3.27.1a/ovidweb.cgi?&S=DPDPFPJMBHDDHOGKNCFKKEIBELHNAA00&SELECT=S.sh%7c&Expand=1&Main+Search+Page=Main+Search+Page) \| [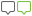](http://ovidsp.tx.ovid.com/sp-3.27.1a/ovidweb.cgi?&S=DPDPFPJMBHDDHOGKNCFKKEIBELHNAA00&R=40&Search+Annotations+Options=SA) \| \|  \| 41 \| (exp animals/ or nonhuman/) not human/ \| 6416042 \| Advanced \| [Display Results](http://ovidsp.tx.ovid.com/sp-3.27.1a/ovidweb.cgi?&S=DPDPFPJMBHDDHOGKNCFKKEIBELHNAA00&SELECT=S.sh%7c&R=41&Process+Action=display)  [More](http://ovidsp.tx.ovid.com/sp-3.27.1a/ovidweb.cgi?&S=DPDPFPJMBHDDHOGKNCFKKEIBELHNAA00&SELECT=S.sh%7c&Expand=1&Main+Search+Page=Main+Search+Page) \| [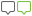](http://ovidsp.tx.ovid.com/sp-3.27.1a/ovidweb.cgi?&S=DPDPFPJMBHDDHOGKNCFKKEIBELHNAA00&R=41&Search+Annotations+Options=SA) \| \|  \| 42 \| 40 not 41 \| 1659059 \| Advanced \| [Display Results](http://ovidsp.tx.ovid.com/sp-3.27.1a/ovidweb.cgi?&S=DPDPFPJMBHDDHOGKNCFKKEIBELHNAA00&SELECT=S.sh%7c&R=42&Process+Action=display)  [More](http://ovidsp.tx.ovid.com/sp-3.27.1a/ovidweb.cgi?&S=DPDPFPJMBHDDHOGKNCFKKEIBELHNAA00&SELECT=S.sh%7c&Expand=1&Main+Search+Page=Main+Search+Page) \| [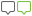](http://ovidsp.tx.ovid.com/sp-3.27.1a/ovidweb.cgi?&S=DPDPFPJMBHDDHOGKNCFKKEIBELHNAA00&R=42&Search+Annotations+Options=SA) \| \|  \| 43 \| 33 and 42 \| 18 \| Advanced \| [Display Results](http://ovidsp.tx.ovid.com/sp-3.27.1a/ovidweb.cgi?&S=DPDPFPJMBHDDHOGKNCFKKEIBELHNAA00&SELECT=S.sh%7c&R=43&Process+Action=display)  [More](http://ovidsp.tx.ovid.com/sp-3.27.1a/ovidweb.cgi?&S=DPDPFPJMBHDDHOGKNCFKKEIBELHNAA00&SELECT=S.sh%7c&Expand=1&Main+Search+Page=Main+Search+Page) \| [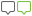](http://ovidsp.tx.ovid.com/sp-3.27.1a/ovidweb.cgi?&S=DPDPFPJMBHDDHOGKNCFKKEIBELHNAA00&R=43&Search+Annotations+Options=SA) \| \|  \| 44 \| 34 and 42 \| 141 \| Advanced \| [Display Results](http://ovidsp.tx.ovid.com/sp-3.27.1a/ovidweb.cgi?&S=DPDPFPJMBHDDHOGKNCFKKEIBELHNAA00&SELECT=S.sh%7c&R=44&Process+Action=display)  [More](http://ovidsp.tx.ovid.com/sp-3.27.1a/ovidweb.cgi?&S=DPDPFPJMBHDDHOGKNCFKKEIBELHNAA00&SELECT=S.sh%7c&Expand=1&Main+Search+Page=Main+Search+Page) \| [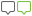](http://ovidsp.tx.ovid.com/sp-3.27.1a/ovidweb.cgi?&S=DPDPFPJMBHDDHOGKNCFKKEIBELHNAA00&R=44&Search+Annotations+Options=SA) \| \|  \| \| \|  \|  \|  \|  \|   Combine with: \| \| \| \| \| \| \| \|  \|  \|  \|  \|  \|  \|  \| \| **CINAHL: (24/11/17)** \|  \|  \|  \|  \|  \|  \| \| \| **Search Terms** \| **Search Options** \| **Actions** \| \| --- \| --- \| --- \| \|  \| S38 \| (S23 OR S24 OR S25 OR S26 OR S27 OR S28 OR S29 OR S30 OR S31 OR S32 OR S33 OR S34 OR S35 OR S36) AND (S22 AND S37) \| Search modes - Boolean/Phrase \| [View Results](javascript:__doPostBack('ctl00$ctl00$MainContentArea$MainContentArea$historyControl$HistoryRepeater$ctl00$linkResults','')) (101)  [View Details](javascript:showShDetails(%22ctl00_ctl00_MainContentArea_MainContentArea_historyControl_ctrlPopup%22,%20%22S38%22);)  [Edit](http://web.b.ebscohost.com/Legacy/Views/UserControls/Ehost/) \| \|  \| S37 \| S23 OR S24 OR S25 OR S26 OR S27 OR S28 OR S29 OR S30 OR S31 OR S32 OR S33 OR S34 OR S35 OR S36 \| Search modes - Boolean/Phrase \| [View Results](javascript:__doPostBack('ctl00$ctl00$MainContentArea$MainContentArea$historyControl$HistoryRepeater$ctl01$linkResults','')) (926,942)  [View Details](javascript:showShDetails(%22ctl00_ctl00_MainContentArea_MainContentArea_historyControl_ctrlPopup%22,%20%22S37%22);)  [Edit](http://web.b.ebscohost.com/Legacy/Views/UserControls/Ehost/) \| \|  \| S36 \| TX allocat* random* \| Search modes - Boolean/Phrase \| [View Results](javascript:__doPostBack('ctl00$ctl00$MainContentArea$MainContentArea$historyControl$HistoryRepeater$ctl02$linkResults','')) (4,952)  [View Details](javascript:showShDetails(%22ctl00_ctl00_MainContentArea_MainContentArea_historyControl_ctrlPopup%22,%20%22S36%22);)  [Edit](http://web.b.ebscohost.com/Legacy/Views/UserControls/Ehost/) \| \|  \| S35 \| (MH "Quantitative Studies") \| Search modes - Boolean/Phrase \| [View Results](javascript:__doPostBack('ctl00$ctl00$MainContentArea$MainContentArea$historyControl$HistoryRepeater$ctl03$linkResults','')) (14,105)  [View Details](javascript:showShDetails(%22ctl00_ctl00_MainContentArea_MainContentArea_historyControl_ctrlPopup%22,%20%22S35%22);)  [Edit](http://web.b.ebscohost.com/Legacy/Views/UserControls/Ehost/) \| \|  \| S34 \| (MH "Placebos") \| Search modes - Boolean/Phrase \| [View Results](javascript:__doPostBack('ctl00$ctl00$MainContentArea$MainContentArea$historyControl$HistoryRepeater$ctl04$linkResults','')) (8,142)  [View Details](javascript:showShDetails(%22ctl00_ctl00_MainContentArea_MainContentArea_historyControl_ctrlPopup%22,%20%22S34%22);)  [Edit](http://web.b.ebscohost.com/Legacy/Views/UserControls/Ehost/) \| \|  \| S33 \| TX placebo* \| Search modes - Boolean/Phrase \| [View Results](javascript:__doPostBack('ctl00$ctl00$MainContentArea$MainContentArea$historyControl$HistoryRepeater$ctl05$linkResults','')) (34,449)  [View Details](javascript:showShDetails(%22ctl00_ctl00_MainContentArea_MainContentArea_historyControl_ctrlPopup%22,%20%22S33%22);)  [Edit](http://web.b.ebscohost.com/Legacy/Views/UserControls/Ehost/) \| \|  \| S32 \| TX random* allocat* \| Search modes - Boolean/Phrase \| [View Results](javascript:__doPostBack('ctl00$ctl00$MainContentArea$MainContentArea$historyControl$HistoryRepeater$ctl06$linkResults','')) (4,952)  [View Details](javascript:showShDetails(%22ctl00_ctl00_MainContentArea_MainContentArea_historyControl_ctrlPopup%22,%20%22S32%22);)  [Edit](http://web.b.ebscohost.com/Legacy/Views/UserControls/Ehost/) \| \|  \| S31 \| (MH "Random Assignment") \| Search modes - Boolean/Phrase \| [View Results](javascript:__doPostBack('ctl00$ctl00$MainContentArea$MainContentArea$historyControl$HistoryRepeater$ctl07$linkResults','')) (35,948)  [View Details](javascript:showShDetails(%22ctl00_ctl00_MainContentArea_MainContentArea_historyControl_ctrlPopup%22,%20%22S31%22);)  [Edit](http://web.b.ebscohost.com/Legacy/Views/UserControls/Ehost/) \| \|  \| S30 \| TX randomi* control* trial* \| Search modes - Boolean/Phrase \| [View Results](javascript:__doPostBack('ctl00$ctl00$MainContentArea$MainContentArea$historyControl$HistoryRepeater$ctl08$linkResults','')) (83,518)  [View Details](javascript:showShDetails(%22ctl00_ctl00_MainContentArea_MainContentArea_historyControl_ctrlPopup%22,%20%22S30%22);)  [Edit](http://web.b.ebscohost.com/Legacy/Views/UserControls/Ehost/) \| \|  \| S29 \| TX ( (singl* n1 blind*) or (singl* n1 mask*) ) \| Search modes - Boolean/Phrase \| [View Results](javascript:__doPostBack('ctl00$ctl00$MainContentArea$MainContentArea$historyControl$HistoryRepeater$ctl09$linkResults','')) (8,813)  [View Details](javascript:showShDetails(%22ctl00_ctl00_MainContentArea_MainContentArea_historyControl_ctrlPopup%22,%20%22S29%22);)  [Edit](http://web.b.ebscohost.com/Legacy/Views/UserControls/Ehost/) \| \|  \| S28 \| TX ( (doubl* n1 blind*) or (doubl* n1 mask*) ) \| Search modes - Boolean/Phrase \| [View Results](javascript:__doPostBack('ctl00$ctl00$MainContentArea$MainContentArea$historyControl$HistoryRepeater$ctl10$linkResults','')) (754,933)  [View Details](javascript:showShDetails(%22ctl00_ctl00_MainContentArea_MainContentArea_historyControl_ctrlPopup%22,%20%22S28%22);)  [Edit](http://web.b.ebscohost.com/Legacy/Views/UserControls/Ehost/) \| \|  \| S27 \| TX ( (tripl* n1 blind*) or (tripl* n1 mask*) ) \| Search modes - Boolean/Phrase \| [View Results](javascript:__doPostBack('ctl00$ctl00$MainContentArea$MainContentArea$historyControl$HistoryRepeater$ctl11$linkResults','')) (169)  [View Details](javascript:showShDetails(%22ctl00_ctl00_MainContentArea_MainContentArea_historyControl_ctrlPopup%22,%20%22S27%22);)  [Edit](http://web.b.ebscohost.com/Legacy/Views/UserControls/Ehost/) \| \|  \| S26 \| TX ( (trebl* n1 blind*) or (trebl* n1 mask*) ) \| Search modes - SmartText Searching \| [View Results](javascript:__doPostBack('ctl00$ctl00$MainContentArea$MainContentArea$historyControl$HistoryRepeater$ctl12$linkResults','')) (153)  [View Details](javascript:showShDetails(%22ctl00_ctl00_MainContentArea_MainContentArea_historyControl_ctrlPopup%22,%20%22S26%22);)  [Edit](http://web.b.ebscohost.com/Legacy/Views/UserControls/Ehost/) \| \|  \| S25 \| TX clinic* n1 trial* \| Search modes - Boolean/Phrase \| [View Results](javascript:__doPostBack('ctl00$ctl00$MainContentArea$MainContentArea$historyControl$HistoryRepeater$ctl13$linkResults','')) (142,707)  [View Details](javascript:showShDetails(%22ctl00_ctl00_MainContentArea_MainContentArea_historyControl_ctrlPopup%22,%20%22S25%22);)  [Edit](http://web.b.ebscohost.com/Legacy/Views/UserControls/Ehost/) \| \|  \| S24 \| PT Clinical trial \| Search modes - Boolean/Phrase \| [View Results](javascript:__doPostBack('ctl00$ctl00$MainContentArea$MainContentArea$historyControl$HistoryRepeater$ctl14$linkResults','')) (54,010)  [View Details](javascript:showShDetails(%22ctl00_ctl00_MainContentArea_MainContentArea_historyControl_ctrlPopup%22,%20%22S24%22);)  [Edit](http://web.b.ebscohost.com/Legacy/Views/UserControls/Ehost/) \| \|  \| S23 \| MH "Clinical Trials+" \| Search modes - Boolean/Phrase \| [View Results](javascript:__doPostBack('ctl00$ctl00$MainContentArea$MainContentArea$historyControl$HistoryRepeater$ctl15$linkResults','')) (150,023)  [View Details](javascript:showShDetails(%22ctl00_ctl00_MainContentArea_MainContentArea_historyControl_ctrlPopup%22,%20%22S23%22);)  [Edit](http://web.b.ebscohost.com/Legacy/Views/UserControls/Ehost/) \| \|  \| S22 \| S15 AND S21 \| Search modes - Boolean/Phrase \| [View Results](javascript:__doPostBack('ctl00$ctl00$MainContentArea$MainContentArea$historyControl$HistoryRepeater$ctl16$linkResults','')) (368)  [View Details](javascript:showShDetails(%22ctl00_ctl00_MainContentArea_MainContentArea_historyControl_ctrlPopup%22,%20%22S22%22);)  [Edit](http://web.b.ebscohost.com/Legacy/Views/UserControls/Ehost/) \| \|  \| S21 \| S16 OR S17 OR S18 OR S19 OR S20 \| Search modes - Boolean/Phrase \| [View Results](javascript:__doPostBack('ctl00$ctl00$MainContentArea$MainContentArea$historyControl$HistoryRepeater$ctl17$linkResults','')) (598,389)  [View Details](javascript:showShDetails(%22ctl00_ctl00_MainContentArea_MainContentArea_historyControl_ctrlPopup%22,%20%22S21%22);)  [Edit](http://web.b.ebscohost.com/Legacy/Views/UserControls/Ehost/) \| \|  \| S20 \| TI ( (child* or infant* or paediatric* or pediatric* or adolescen* or teen* or youth* or boy* or girl* neonat* or baby or babies or babys) ) OR AB ( (child* or infant* or paediatric* or pediatric* or adolescen* or teen* or youth* or boy* or girl* neonat* or baby or babies or babys) ) \| Search modes - Boolean/Phrase \| [View Results](javascript:__doPostBack('ctl00$ctl00$MainContentArea$MainContentArea$historyControl$HistoryRepeater$ctl18$linkResults','')) (364,498)  [View Details](javascript:showShDetails(%22ctl00_ctl00_MainContentArea_MainContentArea_historyControl_ctrlPopup%22,%20%22S20%22);)  [Edit](http://web.b.ebscohost.com/Legacy/Views/UserControls/Ehost/) \| \|  \| S19 \| (MH "Adolescence+") \| Search modes - Boolean/Phrase \| [View Results](javascript:__doPostBack('ctl00$ctl00$MainContentArea$MainContentArea$historyControl$HistoryRepeater$ctl19$linkResults','')) (252,614)  [View Details](javascript:showShDetails(%22ctl00_ctl00_MainContentArea_MainContentArea_historyControl_ctrlPopup%22,%20%22S19%22);)  [Edit](http://web.b.ebscohost.com/Legacy/Views/UserControls/Ehost/) \| \|  \| S18 \| (MH "Infant+") \| Search modes - Boolean/Phrase \| [View Results](javascript:__doPostBack('ctl00$ctl00$MainContentArea$MainContentArea$historyControl$HistoryRepeater$ctl20$linkResults','')) (139,245)  [View Details](javascript:showShDetails(%22ctl00_ctl00_MainContentArea_MainContentArea_historyControl_ctrlPopup%22,%20%22S18%22);)  [Edit](http://web.b.ebscohost.com/Legacy/Views/UserControls/Ehost/) \| \|  \| S17 \| (MH "Child+") \| Search modes - Boolean/Phrase \| [View Results](javascript:__doPostBack('ctl00$ctl00$MainContentArea$MainContentArea$historyControl$HistoryRepeater$ctl21$linkResults','')) (351,474)  [View Details](javascript:showShDetails(%22ctl00_ctl00_MainContentArea_MainContentArea_historyControl_ctrlPopup%22,%20%22S17%22);)  [Edit](http://web.b.ebscohost.com/Legacy/Views/UserControls/Ehost/) \| \|  \| S16 \| (MH "Pediatrics+") \| Search modes - Boolean/Phrase \| [View Results](javascript:__doPostBack('ctl00$ctl00$MainContentArea$MainContentArea$historyControl$HistoryRepeater$ctl22$linkResults','')) (9,480)  [View Details](javascript:showShDetails(%22ctl00_ctl00_MainContentArea_MainContentArea_historyControl_ctrlPopup%22,%20%22S16%22);)  [Edit](http://web.b.ebscohost.com/Legacy/Views/UserControls/Ehost/) \| \|  \| S15 \| S1 OR S2 OR S3 OR S8 OR S9 OR S10 OR S11 OR S12 OR S13 OR S14 \| Search modes - Boolean/Phrase \| [View Results](javascript:__doPostBack('ctl00$ctl00$MainContentArea$MainContentArea$historyControl$HistoryRepeater$ctl23$linkResults','')) (1,410)  [View Details](javascript:showShDetails(%22ctl00_ctl00_MainContentArea_MainContentArea_historyControl_ctrlPopup%22,%20%22S15%22);)  [Edit](http://web.b.ebscohost.com/Legacy/Views/UserControls/Ehost/) \| \|  \| S14 \| TI lannelongue* OR AB lannelongue* \| Search modes - Boolean/Phrase \| [View Results](javascript:__doPostBack('ctl00$ctl00$MainContentArea$MainContentArea$historyControl$HistoryRepeater$ctl24$linkResults','')) (2)  [View Details](javascript:showShDetails(%22ctl00_ctl00_MainContentArea_MainContentArea_historyControl_ctrlPopup%22,%20%22S14%22);)  [Edit](http://web.b.ebscohost.com/Legacy/Views/UserControls/Ehost/) \| \|  \| S13 \| TI apophy* N3 tibia* tuber* OR AB apophy* N3 tibia* tuber* \| Search modes - Boolean/Phrase \| [View Results](javascript:__doPostBack('ctl00$ctl00$MainContentArea$MainContentArea$historyControl$HistoryRepeater$ctl25$linkResults','')) (4)  [View Details](javascript:showShDetails(%22ctl00_ctl00_MainContentArea_MainContentArea_historyControl_ctrlPopup%22,%20%22S13%22);)  [Edit](http://web.b.ebscohost.com/Legacy/Views/UserControls/Ehost/) \| \|  \| S12 \| TI sinding larsen johansson* OR AB sinding larsen johansson* \| Search modes - Boolean/Phrase \| [View Results](javascript:__doPostBack('ctl00$ctl00$MainContentArea$MainContentArea$historyControl$HistoryRepeater$ctl26$linkResults','')) (13)  [View Details](javascript:showShDetails(%22ctl00_ctl00_MainContentArea_MainContentArea_historyControl_ctrlPopup%22,%20%22S12%22);)  [Edit](http://web.b.ebscohost.com/Legacy/Views/UserControls/Ehost/) \| \|  \| S11 \| TI jumpers knee* OR AB jumpers knee* \| Search modes - Boolean/Phrase \| [View Results](javascript:__doPostBack('ctl00$ctl00$MainContentArea$MainContentArea$historyControl$HistoryRepeater$ctl27$linkResults','')) (48)  [View Details](javascript:showShDetails(%22ctl00_ctl00_MainContentArea_MainContentArea_historyControl_ctrlPopup%22,%20%22S11%22);)  [Edit](http://web.b.ebscohost.com/Legacy/Views/UserControls/Ehost/) \| \|  \| S10 \| TI osgood schlatter* OR AB osgood schlatter* \| Search modes - Boolean/Phrase \| [View Results](javascript:__doPostBack('ctl00$ctl00$MainContentArea$MainContentArea$historyControl$HistoryRepeater$ctl28$linkResults','')) (62)  [View Details](javascript:showShDetails(%22ctl00_ctl00_MainContentArea_MainContentArea_historyControl_ctrlPopup%22,%20%22S10%22);)  [Edit](http://web.b.ebscohost.com/Legacy/Views/UserControls/Ehost/) \| \|  \| S9 \| TI ( ((patella* or kneecap* or knee cap*) N3 (tendon* or tendin*)) ) OR AB ( ((patella* or kneecap* or knee cap*) N3 (tendon* or tendin*)) ) \| Search modes - Boolean/Phrase \| [View Results](javascript:__doPostBack('ctl00$ctl00$MainContentArea$MainContentArea$historyControl$HistoryRepeater$ctl29$linkResults','')) (1,168)  [View Details](javascript:showShDetails(%22ctl00_ctl00_MainContentArea_MainContentArea_historyControl_ctrlPopup%22,%20%22S9%22);)  [Edit](http://web.b.ebscohost.com/Legacy/Views/UserControls/Ehost/) \| \|  \| S8 \| S4 AND S7 \| Search modes - Boolean/Phrase \| [View Results](javascript:__doPostBack('ctl00$ctl00$MainContentArea$MainContentArea$historyControl$HistoryRepeater$ctl30$linkResults','')) (68)  [View Details](javascript:showShDetails(%22ctl00_ctl00_MainContentArea_MainContentArea_historyControl_ctrlPopup%22,%20%22S8%22);)  [Edit](http://web.b.ebscohost.com/Legacy/Views/UserControls/Ehost/) \| \|  \| S7 \| S5 OR S6 \| Search modes - Boolean/Phrase \| [View Results](javascript:__doPostBack('ctl00$ctl00$MainContentArea$MainContentArea$historyControl$HistoryRepeater$ctl31$linkResults','')) (2,878)  [View Details](javascript:showShDetails(%22ctl00_ctl00_MainContentArea_MainContentArea_historyControl_ctrlPopup%22,%20%22S7%22);)  [Edit](http://web.b.ebscohost.com/Legacy/Views/UserControls/Ehost/) \| \|  \| S6 \| (MH "Tendon Injuries") \| Search modes - Boolean/Phrase \| [View Results](javascript:__doPostBack('ctl00$ctl00$MainContentArea$MainContentArea$historyControl$HistoryRepeater$ctl32$linkResults','')) (1,754)  [View Details](javascript:showShDetails(%22ctl00_ctl00_MainContentArea_MainContentArea_historyControl_ctrlPopup%22,%20%22S6%22);)  [Edit](http://web.b.ebscohost.com/Legacy/Views/UserControls/Ehost/) \| \|  \| S5 \| (MH "Tendinopathy") \| Search modes - Boolean/Phrase \| [View Results](javascript:__doPostBack('ctl00$ctl00$MainContentArea$MainContentArea$historyControl$HistoryRepeater$ctl33$linkResults','')) (1,231)  [View Details](javascript:showShDetails(%22ctl00_ctl00_MainContentArea_MainContentArea_historyControl_ctrlPopup%22,%20%22S5%22);)  [Edit](http://web.b.ebscohost.com/Legacy/Views/UserControls/Ehost/) \| \|  \| S4 \| (MH "Patella") \| Search modes - Boolean/Phrase \| [View Results](javascript:__doPostBack('ctl00$ctl00$MainContentArea$MainContentArea$historyControl$HistoryRepeater$ctl34$linkResults','')) (1,023)  [View Details](javascript:showShDetails(%22ctl00_ctl00_MainContentArea_MainContentArea_historyControl_ctrlPopup%22,%20%22S4%22);)  [Edit](http://web.b.ebscohost.com/Legacy/Views/UserControls/Ehost/) \| \|  \| S3 \| (MH "Sinding-Larsen-Johansson Syndrome") \| Search modes - Boolean/Phrase \| [View Results](javascript:__doPostBack('ctl00$ctl00$MainContentArea$MainContentArea$historyControl$HistoryRepeater$ctl35$linkResults','')) (10)  [View Details](javascript:showShDetails(%22ctl00_ctl00_MainContentArea_MainContentArea_historyControl_ctrlPopup%22,%20%22S3%22);)  [Edit](http://web.b.ebscohost.com/Legacy/Views/UserControls/Ehost/) \| \|  \| S2 \| (MH "Patellar Ligament") \| Search modes - Boolean/Phrase \| [View Results](javascript:__doPostBack('ctl00$ctl00$MainContentArea$MainContentArea$historyControl$HistoryRepeater$ctl36$linkResults','')) (345)  [View Details](javascript:showShDetails(%22ctl00_ctl00_MainContentArea_MainContentArea_historyControl_ctrlPopup%22,%20%22S2%22);)  [Edit](http://web.b.ebscohost.com/Legacy/Views/UserControls/Ehost/) \| \|  \| S1 \| (MH "Patellar Tendinopathy") \| Search modes - Boolean/Phrase \| [View Results](javascript:__doPostBack('ctl00$ctl00$MainContentArea$MainContentArea$historyControl$HistoryRepeater$ctl37$linkResults','')) (207)  [View Details](javascript:showShDetails(%22ctl00_ctl00_MainContentArea_MainContentArea_historyControl_ctrlPopup%22,%20%22S1%22);)  [Edit](http://web.b.ebscohost.com/Legacy/Views/UserControls/Ehost/) \| \|  \|  \|  \|  \|  \|  \| \| **SPORTDiscus: (24/11/17)** \|  \|  \|  \|  \|  \|  \| \| \| **Search Terms** \| **Search Options** \| **Actions** \| \| --- \| --- \| --- \| \|  \| S23 \| S21 AND S22 \| Search modes - Boolean/Phrase \| [View Results](javascript:__doPostBack('ctl00$ctl00$MainContentArea$MainContentArea$historyControl$HistoryRepeater$ctl00$linkResults','')) (31)  [View Details](javascript:showShDetails(%22ctl00_ctl00_MainContentArea_MainContentArea_historyControl_ctrlPopup%22,%20%22S23%22);)  [Edit](http://web.b.ebscohost.com/Legacy/Views/UserControls/Ehost/) \| \|  \| S22 \| TI ( random* or trial* or control* ) OR AB ( random* or trial* or control* ) \| Search modes - Boolean/Phrase \| [View Results](javascript:__doPostBack('ctl00$ctl00$MainContentArea$MainContentArea$historyControl$HistoryRepeater$ctl01$linkResults','')) (150,439)  [View Details](javascript:showShDetails(%22ctl00_ctl00_MainContentArea_MainContentArea_historyControl_ctrlPopup%22,%20%22S22%22);)  [Edit](http://web.b.ebscohost.com/Legacy/Views/UserControls/Ehost/) \| \|  \| S21 \| S15 AND S20 \| Search modes - Boolean/Phrase \| [View Results](javascript:__doPostBack('ctl00$ctl00$MainContentArea$MainContentArea$historyControl$HistoryRepeater$ctl02$linkResults','')) (191)  [View Details](javascript:showShDetails(%22ctl00_ctl00_MainContentArea_MainContentArea_historyControl_ctrlPopup%22,%20%22S21%22);)  [Edit](http://web.b.ebscohost.com/Legacy/Views/UserControls/Ehost/) \| \|  \| S20 \| S16 OR S17 OR S18 OR S19 \| Search modes - Boolean/Phrase \| [View Results](javascript:__doPostBack('ctl00$ctl00$MainContentArea$MainContentArea$historyControl$HistoryRepeater$ctl03$linkResults','')) (149,564)  [View Details](javascript:showShDetails(%22ctl00_ctl00_MainContentArea_MainContentArea_historyControl_ctrlPopup%22,%20%22S20%22);)  [Edit](http://web.b.ebscohost.com/Legacy/Views/UserControls/Ehost/) \| \|  \| S19 \| TI ( (child* or infant* or paediatric* or pediatric* or adolescen* or teen* or youth* or boy* or girl* neonat* or baby or babies or babys) ) OR AB ( (child* or infant* or paediatric* or pediatric* or adolescen* or teen* or youth* or boy* or girl* neonat* or baby or babies or babys) ) \| Search modes - Boolean/Phrase \| [View Results](javascript:__doPostBack('ctl00$ctl00$MainContentArea$MainContentArea$historyControl$HistoryRepeater$ctl04$linkResults','')) (123,580)  [View Details](javascript:showShDetails(%22ctl00_ctl00_MainContentArea_MainContentArea_historyControl_ctrlPopup%22,%20%22S19%22);)  [Edit](http://web.b.ebscohost.com/Legacy/Views/UserControls/Ehost/) \| \|  \| S18 \| DE "TEENAGERS" \| Search modes - Boolean/Phrase \| [View Results](javascript:__doPostBack('ctl00$ctl00$MainContentArea$MainContentArea$historyControl$HistoryRepeater$ctl05$linkResults','')) (28,564)  [View Details](javascript:showShDetails(%22ctl00_ctl00_MainContentArea_MainContentArea_historyControl_ctrlPopup%22,%20%22S18%22);)  [Edit](http://web.b.ebscohost.com/Legacy/Views/UserControls/Ehost/) \| \|  \| S17 \| DE "CHILDREN" \| Search modes - Boolean/Phrase \| [View Results](javascript:__doPostBack('ctl00$ctl00$MainContentArea$MainContentArea$historyControl$HistoryRepeater$ctl06$linkResults','')) (36,686)  [View Details](javascript:showShDetails(%22ctl00_ctl00_MainContentArea_MainContentArea_historyControl_ctrlPopup%22,%20%22S17%22);)  [Edit](http://web.b.ebscohost.com/Legacy/Views/UserControls/Ehost/) \| \|  \| S16 \| DE "PEDIATRICS" \| Search modes - Boolean/Phrase \| [View Results](javascript:__doPostBack('ctl00$ctl00$MainContentArea$MainContentArea$historyControl$HistoryRepeater$ctl07$linkResults','')) (1,050)  [View Details](javascript:showShDetails(%22ctl00_ctl00_MainContentArea_MainContentArea_historyControl_ctrlPopup%22,%20%22S16%22);)  [Edit](http://web.b.ebscohost.com/Legacy/Views/UserControls/Ehost/) \| \|  \| S15 \| S1 OR S6 OR S7 OR S8 OR S9 OR S10 OR S11 OR S12 OR S13 OR S14 \| Search modes - Boolean/Phrase \| [View Results](javascript:__doPostBack('ctl00$ctl00$MainContentArea$MainContentArea$historyControl$HistoryRepeater$ctl08$linkResults','')) (2,619)  [View Details](javascript:showShDetails(%22ctl00_ctl00_MainContentArea_MainContentArea_historyControl_ctrlPopup%22,%20%22S15%22);)  [Edit](http://web.b.ebscohost.com/Legacy/Views/UserControls/Ehost/) \| \|  \| S14 \| TI lannelongue* OR AB lannelongue* \| Search modes - Boolean/Phrase \| [View Results](javascript:__doPostBack('ctl00$ctl00$MainContentArea$MainContentArea$historyControl$HistoryRepeater$ctl09$linkResults','')) (1)  [View Details](javascript:showShDetails(%22ctl00_ctl00_MainContentArea_MainContentArea_historyControl_ctrlPopup%22,%20%22S14%22);)  [Edit](http://web.b.ebscohost.com/Legacy/Views/UserControls/Ehost/) \| \|  \| S13 \| TI (apophy* N3 tibia* tuber*) OR AB (apophy* N3 tibia* tuber*) \| Search modes - Boolean/Phrase \| [View Results](javascript:__doPostBack('ctl00$ctl00$MainContentArea$MainContentArea$historyControl$HistoryRepeater$ctl10$linkResults','')) (6)  [View Details](javascript:showShDetails(%22ctl00_ctl00_MainContentArea_MainContentArea_historyControl_ctrlPopup%22,%20%22S13%22);)  [Edit](http://web.b.ebscohost.com/Legacy/Views/UserControls/Ehost/) \| \|  \| S12 \| TI jumpers knee* OR AB jumpers knee* \| Search modes - Boolean/Phrase \| [View Results](javascript:__doPostBack('ctl00$ctl00$MainContentArea$MainContentArea$historyControl$HistoryRepeater$ctl11$linkResults','')) (209)  [View Details](javascript:showShDetails(%22ctl00_ctl00_MainContentArea_MainContentArea_historyControl_ctrlPopup%22,%20%22S12%22);)  [Edit](http://web.b.ebscohost.com/Legacy/Views/UserControls/Ehost/) \| \|  \| S11 \| TI sinding larsen johansson* OR AB sinding larsen johansson* \| Search modes - Boolean/Phrase \| [View Results](javascript:__doPostBack('ctl00$ctl00$MainContentArea$MainContentArea$historyControl$HistoryRepeater$ctl12$linkResults','')) (24)  [View Details](javascript:showShDetails(%22ctl00_ctl00_MainContentArea_MainContentArea_historyControl_ctrlPopup%22,%20%22S11%22);)  [Edit](http://web.b.ebscohost.com/Legacy/Views/UserControls/Ehost/) \| \|  \| S10 \| TI osgood schlatter* OR AB osgood schlatter* \| Search modes - Boolean/Phrase \| [View Results](javascript:__doPostBack('ctl00$ctl00$MainContentArea$MainContentArea$historyControl$HistoryRepeater$ctl13$linkResults','')) (150)  [View Details](javascript:showShDetails(%22ctl00_ctl00_MainContentArea_MainContentArea_historyControl_ctrlPopup%22,%20%22S10%22);)  [Edit](http://web.b.ebscohost.com/Legacy/Views/UserControls/Ehost/) \| \|  \| S9 \| TI ( (patella* or kneecap* or knee cap*) N3 (tendon* or tendin*) ) OR AB ( (patella* or kneecap* or knee cap*) N3 (tendon* or tendin*) ) \| Search modes - Boolean/Phrase \| [View Results](javascript:__doPostBack('ctl00$ctl00$MainContentArea$MainContentArea$historyControl$HistoryRepeater$ctl14$linkResults','')) (2,234)  [View Details](javascript:showShDetails(%22ctl00_ctl00_MainContentArea_MainContentArea_historyControl_ctrlPopup%22,%20%22S9%22);)  [Edit](http://web.b.ebscohost.com/Legacy/Views/UserControls/Ehost/) \| \|  \| S8 \| DE "OSGOOD-Schlatter disease" \| Search modes - Boolean/Phrase \| [View Results](javascript:__doPostBack('ctl00$ctl00$MainContentArea$MainContentArea$historyControl$HistoryRepeater$ctl15$linkResults','')) (21)  [View Details](javascript:showShDetails(%22ctl00_ctl00_MainContentArea_MainContentArea_historyControl_ctrlPopup%22,%20%22S8%22);)  [Edit](http://web.b.ebscohost.com/Legacy/Views/UserControls/Ehost/) \| \|  \| S7 \| DE "PATELLAR ligament" \| Search modes - Boolean/Phrase \| [View Results](javascript:__doPostBack('ctl00$ctl00$MainContentArea$MainContentArea$historyControl$HistoryRepeater$ctl16$linkResults','')) (204)  [View Details](javascript:showShDetails(%22ctl00_ctl00_MainContentArea_MainContentArea_historyControl_ctrlPopup%22,%20%22S7%22);)  [Edit](http://web.b.ebscohost.com/Legacy/Views/UserControls/Ehost/) \| \|  \| S6 \| S2 AND S5 \| Search modes - Boolean/Phrase \| [View Results](javascript:__doPostBack('ctl00$ctl00$MainContentArea$MainContentArea$historyControl$HistoryRepeater$ctl17$linkResults','')) (15)  [View Details](javascript:showShDetails(%22ctl00_ctl00_MainContentArea_MainContentArea_historyControl_ctrlPopup%22,%20%22S6%22);)  [Edit](http://web.b.ebscohost.com/Legacy/Views/UserControls/Ehost/) \| \|  \| S5 \| S3 OR S4 \| Search modes - Boolean/Phrase \| [View Results](javascript:__doPostBack('ctl00$ctl00$MainContentArea$MainContentArea$historyControl$HistoryRepeater$ctl18$linkResults','')) (1,128)  [View Details](javascript:showShDetails(%22ctl00_ctl00_MainContentArea_MainContentArea_historyControl_ctrlPopup%22,%20%22S5%22);)  [Edit](http://web.b.ebscohost.com/Legacy/Views/UserControls/Ehost/) \| \|  \| S4 \| DE "TENDON injuries" OR DE "ACHILLES tendon injuries" \| Search modes - Boolean/Phrase \| [View Results](javascript:__doPostBack('ctl00$ctl00$MainContentArea$MainContentArea$historyControl$HistoryRepeater$ctl19$linkResults','')) (979)  [View Details](javascript:showShDetails(%22ctl00_ctl00_MainContentArea_MainContentArea_historyControl_ctrlPopup%22,%20%22S4%22);)  [Edit](http://web.b.ebscohost.com/Legacy/Views/UserControls/Ehost/) \| \|  \| S3 \| DE "TENDINOSIS" \| Search modes - Boolean/Phrase \| [View Results](javascript:__doPostBack('ctl00$ctl00$MainContentArea$MainContentArea$historyControl$HistoryRepeater$ctl20$linkResults','')) (166)  [View Details](javascript:showShDetails(%22ctl00_ctl00_MainContentArea_MainContentArea_historyControl_ctrlPopup%22,%20%22S3%22);)  [Edit](http://web.b.ebscohost.com/Legacy/Views/UserControls/Ehost/) \| \|  \| S2 \| DE "PATELLA" OR DE "PATELLOFEMORAL joint" \| Search modes - Boolean/Phrase \| [View Results](javascript:__doPostBack('ctl00$ctl00$MainContentArea$MainContentArea$historyControl$HistoryRepeater$ctl21$linkResults','')) (2,268)  [View Details](javascript:showShDetails(%22ctl00_ctl00_MainContentArea_MainContentArea_historyControl_ctrlPopup%22,%20%22S2%22);)  [Edit](http://web.b.ebscohost.com/Legacy/Views/UserControls/Ehost/) \| \|  \| S1 \| DE "JUMPER'S knee" \| Search modes - Boolean/Phrase \| [View Results](javascript:__doPostBack('ctl00$ctl00$MainContentArea$MainContentArea$historyControl$HistoryRepeater$ctl22$linkResults','')) (319)  [View Details](javascript:showShDetails(%22ctl00_ctl00_MainContentArea_MainContentArea_historyControl_ctrlPopup%22,%20%22S1%22);)  [Edit](http://web.b.ebscohost.com/Legacy/Views/UserControls/Ehost/) \| \|  \|  \|  \|  \|  \|  \| \|  \|  \|  \|  \|  \|  \|  \| \|  \|  \|  \|  \|  \|  \|  \| \|  \|  \|  \|  \|  \|  \|  \| \|  \|  \|  \|  \|  \|  \|  \| \|  \|  \|  \|  \|  \|  \|  \| |  |  |  |  |  |  |
| --- | --- | --- | --- | --- | --- | --- | --- | --- | --- | --- | --- | --- | --- | --- | --- | --- | --- | --- | --- | --- | --- | --- | --- | --- | --- | --- | --- | --- | --- | --- | --- | --- | --- | --- | --- | --- | --- | --- | --- | --- | --- | --- | --- | --- | --- | --- | --- | --- | --- | --- | --- | --- | --- | --- | --- | --- | --- | --- | --- | --- | --- | --- | --- | --- | --- | --- | --- | --- | --- | --- | --- | --- | --- | --- | --- | --- | --- | --- | --- | --- | --- | --- | --- | --- | --- | --- | --- | --- | --- | --- | --- | --- | --- | --- | --- | --- | --- | --- | --- | --- | --- | --- | --- | --- | --- | --- | --- | --- | --- | --- | --- | --- | --- | --- | --- | --- | --- | --- | --- | --- | --- | --- | --- | --- | --- | --- | --- | --- | --- | --- | --- | --- | --- | --- | --- | --- | --- | --- | --- | --- | --- | --- | --- | --- | --- | --- | --- | --- | --- | --- | --- | --- | --- | --- | --- | --- | --- | --- | --- | --- | --- | --- | --- | --- | --- | --- | --- | --- | --- | --- | --- | --- | --- | --- | --- | --- | --- | --- | --- | --- | --- | --- | --- | --- | --- | --- | --- | --- | --- | --- | --- | --- | --- | --- | --- | --- | --- | --- | --- | --- | --- | --- | --- | --- | --- | --- | --- | --- | --- | --- | --- | --- | --- | --- | --- | --- | --- | --- | --- | --- | --- | --- | --- | --- | --- | --- | --- | --- | --- | --- | --- | --- | --- | --- | --- | --- | --- | --- | --- | --- | --- | --- | --- | --- | --- | --- | --- | --- | --- | --- | --- | --- | --- | --- | --- | --- | --- | --- | --- | --- | --- | --- | --- | --- | --- | --- | --- | --- | --- | --- | --- | --- | --- | --- | --- | --- | --- | --- | --- | --- | --- | --- | --- | --- | --- | --- | --- | --- | --- | --- | --- | --- | --- | --- | --- | --- | --- | --- | --- | --- | --- | --- | --- | --- | --- | --- | --- | --- | --- | --- | --- | --- | --- | --- | --- | --- | --- | --- | --- | --- | --- | --- | --- | --- | --- | --- | --- | --- | --- | --- | --- | --- | --- | --- | --- | --- | --- | --- | --- | --- | --- | --- | --- | --- | --- | --- | --- | --- | --- | --- | --- | --- | --- | --- | --- | --- | --- | --- | --- | --- | --- | --- | --- | --- | --- | --- | --- | --- | --- | --- | --- | --- | --- | --- | --- | --- | --- | --- | --- | --- | --- | --- | --- | --- | --- | --- | --- | --- | --- | --- | --- | --- | --- | --- | --- | --- | --- | --- | --- | --- | --- | --- | --- | --- | --- | --- | --- | --- | --- | --- | --- | --- | --- | --- | --- | --- | --- | --- | --- | --- | --- | --- | --- | --- | --- | --- | --- | --- | --- | --- | --- | --- | --- | --- | --- | --- | --- | --- | --- | --- | --- | --- | --- | --- | --- | --- | --- | --- | --- | --- | --- | --- | --- | --- | --- | --- | --- | --- | --- | --- | --- | --- | --- | --- | --- | --- | --- | --- | --- | --- | --- | --- | --- | --- | --- | --- | --- | --- | --- | --- | --- | --- | --- | --- | --- | --- | --- | --- | --- | --- | --- | --- | --- | --- | --- | --- | --- | --- | --- | --- | --- | --- | --- | --- | --- | --- | --- | --- | --- | --- | --- | --- | --- | --- | --- | --- | --- | --- | --- | --- | --- | --- | --- | --- | --- | --- | --- | --- | --- | --- | --- | --- | --- | --- | --- | --- | --- | --- | --- | --- | --- | --- | --- | --- | --- | --- | --- | --- | --- | --- | --- | --- | --- | --- | --- | --- | --- | --- | --- | --- | --- | --- | --- | --- | --- | --- | --- | --- | --- | --- | --- | --- | --- | --- | --- | --- | --- | --- | --- | --- | --- | --- | --- | --- | --- | --- | --- | --- | --- | --- | --- | --- | --- | --- | --- | --- | --- | --- | --- | --- | --- | --- | --- | --- | --- | --- | --- | --- | --- | --- | --- | --- | --- | --- | --- | --- | --- | --- | --- | --- | --- | --- | --- | --- | --- | --- | --- | --- | --- | --- | --- | --- | --- | --- | --- | --- | --- | --- | --- | --- | --- | --- | --- | --- | --- | --- | --- | --- | --- | --- | --- | --- | --- | --- | --- | --- | --- | --- | --- | --- | --- | --- | --- | --- | --- | --- | --- | --- | --- | --- | --- | --- | --- | --- | --- | --- | --- | --- | --- | --- | --- | --- | --- | --- | --- | --- | --- | --- | --- | --- | --- | --- | --- | --- | --- | --- | --- | --- | --- | --- | --- | --- | --- | --- | --- | --- | --- | --- | --- | --- | --- | --- | --- | --- | --- | --- | --- | --- | --- | --- | --- | --- | --- | --- | --- | --- | --- | --- | --- | --- | --- | --- | --- | --- | --- | --- | --- | --- | --- | --- | --- | --- | --- | --- | --- | --- | --- | --- | --- | --- | --- | --- | --- | --- | --- | --- | --- | --- | --- | --- | --- | --- | --- | --- | --- | --- | --- | --- | --- | --- | --- | --- | --- | --- | --- | --- | --- | --- | --- | --- | --- | --- | --- | --- | --- | --- | --- | --- | --- | --- | --- | --- | --- | --- | --- | --- | --- | --- | --- | --- | --- | --- | --- | --- | --- | --- | --- | --- | --- | --- | --- | --- | --- | --- | --- | --- | --- | --- | --- | --- | --- | --- | --- | --- | --- | --- | --- | --- | --- | --- | --- | --- | --- | --- | --- | --- | --- | --- | --- | --- | --- | --- | --- | --- | --- | --- | --- | --- | --- | --- | --- | --- | --- | --- | --- | --- | --- | --- | --- | --- | --- | --- | --- | --- | --- | --- | --- | --- | --- | --- | --- | --- | --- | --- | --- | --- | --- | --- | --- | --- | --- | --- | --- | --- | --- | --- | --- | --- | --- | --- | --- | --- | --- | --- | --- | --- | --- | --- | --- | --- | --- | --- | --- | --- | --- | --- | --- | --- | --- | --- | --- | --- | --- | --- | --- | --- | --- | --- | --- | --- | --- | --- | --- | --- | --- | --- | --- | --- | --- | --- | --- | --- | --- | --- | --- | --- | --- | --- | --- | --- | --- | --- | --- | --- | --- | --- | --- | --- | --- | --- | --- | --- | --- | --- | --- | --- | --- | --- | --- | --- | --- | --- | --- | --- |
|  | | | | | | |
|  |  |  |  |  |  |  |
|  |  |  |  |  |  |  |
|  |  |  |  |  |  |  |
|  |  |  |  |  |  |  |
|  |  |  |  |  |  |  |
|  |  |  |  |  |  |  |
|  |  |  |  |  |  |  |
|  |  |  |  |  |  |  |
|  |  |  |  |  |  |  |
