# Supplementary material for: Therapeutic interventions in children and adolescents with patellar tendon related pain: a systematic review
Source: BMJ Open Sport Exerc Med. 2018 Aug 13;4(1):e000383. doi: 10.1136/bmjsem-2018-000383 (PMC6109948; doi:10.1136/bmjsem-2018-000383)
Supplement: Supplementary data [file bmjsem-2018-000383supp002.docx]

**Characteristics of studies**

**Characteristics of included studies**

**Biernat 2014**

| **Methods** | **Study design:** Randomized controlled trial  **Study grouping:** Parallel group |
| --- | --- |
| **Participants** | **Baseline Characteristics**  Control   - *age (years)*: 16.5 (0.8) - *body mass (kg)*: 71.5 (5.8) - *height*: 184.8 (3.9) - *body fat %*: 14.3 (3)   Experimental   - *age (years)*: 17.2(0/6) - *body mass (kg)*: 80.1 (10.1) - *height*: 192.9 (5.3) - *body fat %*: 14.7 (3)   Overall   - *age (years)*: - *body mass (kg)*: - *height*: - *body fat %*:   **Included criteria:** Volleyball playersPatellar tendinopathy although unclear how diagnosis defined  **Excluded criteria:** Not specified  **Pretreatment:** - |
| **Interventions** | **Intervention Characteristics**  Control   - *Specific exercises*: No specific exercises - *General advice* : Continued competitive activity and functional exercises   Experimental   - *Specific exercises*: The key element of our rehabilitation protocol was eccentric squat on decline board .The inclination angle was 25 degrees. During the squat, the eccentric phase (lowering the center of the body mass) was done on 1 lower limb to the angle 60 degrees of flexion in the knee joint similarly as in the protocol applied by Zwerver. The concentric phase of the squat (elevation of the body mass center) was done bilaterally to erect position. The tested athlete kept the trunk straight to limit influence of gluteus maximus. Inclination of the board limits the work of plantar flexors. The players from the experimental group done the squats once a day, on the left and the right legs, in 3 series,15 repetitions each. On the days when the volleyball players took part in matches or had intense trainings, the eccentric squats were not done. An extra element during eccentric squats introduced in the fourth week of the program was unstable surface, which increased the requirements of body stabilization and caused rotation in the knee joint during the squat - *General advice* : Continued competitive activity and functional exercises |
| **Outcomes** | *VISA-P questionnaire*   - **Outcome type**: ContinuousOutcome - **Data value**: Endpoint   *Jump height*   - **Outcome type**: ContinuousOutcome - **Data value**: Endpoint   *Maximal power*   - **Outcome type**: ContinuousOutcome - **Data value**: Endpoint |
| **Identification** | **Sponsorship source:** No sponsorship  **Country:** Poland  **Setting:** University Physical Therapy  **Comments:** -  **Authors name:** Dr. Łukasz Trzaskoma  **Institution:** he Jozef Pilsudski University of Physical Education in Warsaw  **Email:** lukasz@tf.hu  **Address:** The Jozef Pilsudski University of Physical Education in Warsaw,Warsaw, Poland |
| **Notes** | *Benjamin Dean* on 25/12/2017 00:39 **Included** request to author 24/12/17  *Benjamin Dean* on 18/01/2018 21:44 **Included** dariusz.czaprowski@interia.pl emailed |

**Risk of bias table**

| **Bias** | **Authors' judgement** | **Support for judgement** |
| --- | --- | --- |
| Random sequence generation (selection bias) |  | Judgement Comment: Not mentioned as to how. |
| Allocation concealment (selection bias) |  | Unclear |
| Blinding of participants and personnel (performance bias) |  | Judgement Comment: Participants not blinded. |
| Blinding of outcome assessment (detection bias) |  | Unclear |
| Incomplete outcome data (attrition bias) |  | Quote: "Fifteen players from the experimental group (E) and 13 from the control group (C) ﬁnished the tests." |
| Selective reporting (reporting bias) |  | Judgement Comment: No primary outcome defined. Multiple outcomes. No protocol specified. |
| Other bias |  |  |

**Cannell 2001**

| **Methods** | **Study design:** Randomized controlled trial  **Study grouping:** Parallel group |
| --- | --- |
| **Participants** | **Baseline Characteristics**  Drop squat   - *Sex: males :females*: 7:3 - *Age (years)*: 26 (23 to 29) - *Weight (kg)*: 76 (69 to 83) - *Height (cm)*: 172 (165 to 179) - *Duration of symptoms*: 3.1 (1.6 to 4.6)   Leg extension/curl   - *Sex: males :females*: 6:3 - *Age (years)*: 26 (19 to 33) - *Weight (kg)*: 74 (66 to 82) - *Height (cm)*: 174 (167 to 181) - *Duration of symptoms*: 4.2 (2.3 to 6.1)   Overall   - *Sex: males :females*: - *Age (years)*: - *Weight (kg)*: - *Height (cm)*: - *Duration of symptoms*:   **Included criteria:** Clinical diagnosis of jumper's knee - (a) sports related anterior knee pain well localised to the inferior pole of the patella and present for more than four weeks, (b) no other knee symptoms currently or previous to this episode of jumper’s knee, (c)moderate or severe tenderness to palpation at the patellar tendon insertion into the patella,and (d) otherwise normal knee examination.  **Excluded criteria:** Use of orthotics  **Pretreatment:** |
| **Interventions** | **Intervention Characteristics**  Drop squat   - *Exercises*: The squat group performed three sets of 20 drops once a day five days a week. The drop squats were performed by subjects starting from a standing position and unlocking their knees rapidly and dropping until both thighs were just short of parallel to the ground.Subjects were advised to use the quadriceps muscles of both legs to stop their fall just short of the thighs being parallel to the ground. The aim of the drop squats was to obtain high eccentric loading. When the subject was able to do three sets of 20 drops easily, he/she progressed to the next level of work as shown in table 1. Subjects were encouraged to ice their patellar tendons after performing the drop squat programme and to expect some pain.Once the subject’s knee pain was completely absent, he/she began an alternate day running programme beginning with 1 km in running athletes and increasing by 1 km every third run,with intensity increasing as shown in table 1. - *General advice*: Subjects in both groups were treated with ice, anti-inflammatory medications, and relative rest in the first two weeks of the study.   Leg extension/curl   - *Exercises*: The leg extension/curl group performed three sets of 10 lifts for each of the leg extension and leg curl exercises, once a day five days a week. For leg extension, subjects were advised to slowly lift the weight with the injured leg to full extension and to hold the weight in that position for two seconds. Each lift from start to finish took at least five seconds. This lift was repeated up to 10 times (one set) before the subject rested. Subjects began with a 5kg weight and gradually increased their repetitions until they could do three sets of 10 with that weight. Once this was achieved, subjects progressed according to the protocol in table 2.The aim of the leg extension exercise, and also the leg curl described below, was to provide high concentric loading.Subjects performed the isotonic hamstring curl with half of the weight used for leg extension (table 2). The subject lay prone on the table and slowly flexed the knee to 90°, held the knee flexed in this position for two seconds,and then slowly returned the weight to the starting position. Each lift from start to finish took at least five seconds. Subjects completed three sets of 10 repetitions with the same weight on each leg, once a day five days a week.Once the subject’s knee pain was completely absent, he/she began an alternate day running programme beginning with 1 km in running athletes and increasing by 1 km every third run,with intensity increasing as shown in table 2. - *General advice*: Subjects in both groups were treated with ice, anti-inflammatory medications, and relative rest in the first two weeks of the study. |
| **Outcomes** | *Pain score*   - **Outcome type**: ContinuousOutcome - **Data value**: Endpoint   *Quadriceps moment of force*   - **Outcome type**: ContinuousOutcome - **Data value**: Endpoint   *Hamstring moment of force*   - **Outcome type**: ContinuousOutcome - **Data value**: Endpoint |
| **Identification** | **Sponsorship source:** None  **Country:** Canada  **Setting:** The Allan McGavin Sports Medicine Centre and School of Human Kinetics,University of British Columbia, Vancouver, Canada  **Comments:**  **Authors name:** Karim Khan  **Institution:** The Allan McGavin Sports Medicine Centre and School of Human Kinetics,University of British Columbia, Vancouver, Canada  **Email:** kkhan@unixg.ubc.ca  **Address:** |
| **Notes** | *Benjamin Dean* on 25/12/2017 01:34 **Included** note sent 24/12/17  *Benjamin Dean* on 18/01/2018 21:38 **Included** second email sent |

**Risk of bias table**

| **Bias** | **Authors' judgement** | **Support for judgement** |
| --- | --- | --- |
| Random sequence generation (selection bias) |  | Quote: "Subjects were randomised by sealed envelope"  Judgement Comment: randomised allocation |
| Allocation concealment (selection bias) |  | Judgement Comment: Sealed envelopes. |
| Blinding of participants and personnel (performance bias) |  | Judgement Comment: Subjects not blinded as had to know which exercises they were performing. |
| Blinding of outcome assessment (detection bias) |  | Quote: "Research assistants blinded to subjects’ treat- ment group collected data."  Judgement Comment: researchers were blinded to the data collected about the treatment group |
| Incomplete outcome data (attrition bias) |  |  |
| Selective reporting (reporting bias) |  | Quote: "Primary outcome measures were pain (visual analogue scale 1–10) and return to sport. Secondary outcome measures included quadriceps and ham- string moment of force using a Cybex II isokinetic dynamometer at 30°/second."  Judgement Comment: Two primary outcomes used. |
| Other bias |  |  |

**Jonsson 2005**

| **Methods** | **Study design:** Randomized controlled trial  **Study grouping:** Parallel group |
| --- | --- |
| **Participants** | **Baseline Characteristics**  Concentric   - *age, years*: 24.1 +/- 6.4 - *height, cm*: 180.5 +/- 4.6 - *weight, kg*: 79.5 +/- 5.3 - *duration of symptoms, months*: 19.6 +/- 20.3   Eccentric   - *age, years*: 25.7 +/- 9.9 - *height, cm*: 176.6 +/- 6.6 - *weight, kg*: 74.8 +/- 7.8 - *duration of symptoms, months*: 15.4 +/- 6   Overall   - *age, years*: - - *height, cm*: - - *weight, kg*: - - *duration of symptoms, months*: -   **Included criteria:** Pain in the proximal patellar tendon during or after patellar tendon loading activity, tenderness in the proximal patellar tendon during palpation, and structural tendon changes together with neovascularisation in the proximal patellar tendon at US and colour Doppler examination. US was performed with a linear transducer  **Excluded criteria:** A history of patello-femoral pain, surgical treatment of the patellar tendon, and knee arthrosis  **Pretreatment:** - |
| **Interventions** | **Intervention Characteristics**  Concentric   - *Specific exercises*: concentric quadriceps training while standing on a decline board. - *General treatment*: Both training groups were given careful instructions by the same physiotherapist (PJ) on how to perform the training and increase the load. Both practical and hand written instructions were given. The training programme consisted of three sets of 15 repetitions each,performed twice a day, 7 days a week, for 12 weeks. The patients were told that muscle soreness during the first 1–2 weeks was to be expected. The training was supposed to be painful, and when there was no pain in the patellar tendon during training, the load was to be increased to reach a new level of painful training by gradually add weights to a backpack. The patients themselves decided how much pain was acceptable. The patients were followed up by the physiotherapist (PJ) after 6 weeks of training. If there was no severe pain, the patients were told to start sport specific training and gradually return to their previous (before injury) sporting activity.For both the eccentric and concentric training, the patients were instructed to keep the trunk in an upright position to minimise gluteal muscle activity while standing on a 25 ̊decline board. The purpose was to relax the calf muscles in order to increase the demands of the knee extensor muscles. If the patient had bilateral jumper’s knee, the programme was completed separately for each leg.   Eccentric   - *Specific exercises*: eccentric quadriceps training while standing on a decline board - *General treatment*: Both training groups were given careful instructions by the same physiotherapist (PJ) on how to perform the training and increase the load. Both practical and hand written instructions were given. The training programme consisted of three sets of 15 repetitions each,performed twice a day, 7 days a week, for 12 weeks. The patients were told that muscle soreness during the first 1–2 weeks was to be expected. The training was supposed to be painful, and when there was no pain in the patellar tendon during training, the load was to be increased to reach a new level of painful training by gradually add weights to a backpack. The patients themselves decided how much pain was acceptable. The patients were followed up by the physiotherapist (PJ) after 6 weeks of training. If there was no severe pain, the patients were told to start sport specific training and gradually return to their previous (before injury) sporting activity.For both the eccentric and concentric training, the patients were instructed to keep the trunk in an upright position to minimise gluteal muscle activity while standing on a 25 ̊decline board. The purpose was to relax the calf muscles in order to increase the demands of the knee extensor muscles. If the patient had bilateral jumper’s knee, the programme was completed separately for each leg. |
| **Outcomes** | *VISA*   - **Outcome type**: ContinuousOutcome - **Reporting**: Fully reported - **Data value**: Endpoint   *Pain scores (VAS)*   - **Outcome type**: ContinuousOutcome - **Data value**: Endpoint |
| **Identification** | **Sponsorship source:** None  **Country:** Sweden  **Setting:** Sports Medicine Unit  **Comments:** -  **Authors name:** Per Jonsson  **Institution:** Sports Medicine Unit, University of Umea  **Email:** per.jonsson@idrott.umu.se  **Address:** S-901 87 Umea, Sweden |
| **Notes** | *Benjamin Dean* on 25/12/2017 00:34 **Included** requested data from author 24/12/17  *Benjamin Dean* on 26/12/2017 23:12 **Included** not possible to get data (author email) |

**Risk of bias table**

| **Bias** | **Authors' judgement** | **Support for judgement** |
| --- | --- | --- |
| Random sequence generation (selection bias) |  | Quote: " <b>Randomisation procedure Sample size/power analysis It was calculated that 20 patients in each group were needed for there to be an 80% chance of detecting a difference in the results of the treatments at the 0.05 significance level. The patients were randomly allocated to either eccentric or concentric quadriceps training while standing on a decline board.</b> Treatment models There were two"  Judgement Comment: Not clarified how. |
| Allocation concealment (selection bias) |  | Judgement Comment: Not described. |
| Blinding of participants and personnel (performance bias) |  | Judgement Comment: Not described. |
| Blinding of outcome assessment (detection bias) |  | Unclear |
| Incomplete outcome data (attrition bias) |  |  |
| Selective reporting (reporting bias) |  | Judgement Comment: No trial protocol published/cited. |
| Other bias |  |  |

**Topol 2011**

| **Methods** | **Study design:** Randomized controlled trial  **Study grouping:** Parallel group |
| --- | --- |
| **Participants** | **Baseline Characteristics**  Local anaesthetic  Dextrose and local anaesthetic  Usual care  Overall  **Included criteria:** Girls age 9 to 15 and boys aged 10 to 17in the area of Rosario, Argentina, were screened for anterior knee pain, but only if they were involved in a jumping or kicking sport on an organized team with a coach.Reproduction of the exact pain and localization of pain precisely to the tibial tuberosity during a single leg squat.Once confident of the diagnosis, patients were required to have at-tempted at least 2 months of formal and gently progressive hamstring stretching, quads strengthening, gradual sport reintroduction, and to have had pain with sport for at least 3 months.  **Excluded criteria:** Patellofemoral crepitus or patellar origin tenderness  **Pretreatment:** - |
| **Interventions** | **Intervention Characteristics**  Local anaesthetic   - *Injection*: 1% lidocaine - *General treatment after injections*: Acetaminophen was advised if neededfor postinjection discomfort. Athletes were advised not to run or kick for 1week after the first injection, and to run as tolerated after the first week.They were advised not to run or kick for 3 days after both the second and any subsequent injections. Usually,they started playing sports with com-petition if doing well after the second injection. Note that many athletes with an NPPS score of 3 were used to play-ing with pain; these athletes were encouraged to engage in a sporting activity only if the activity was not accompanied or followed by pain during the period of treatment.As an incentive for study participation,and to potentially avoid sports drop-out, all study participants that did not reach an NPPS score of 0 could choose to receive dextrose injection after 3 months (the point at which the actual injectant was revealed to the treating physician and patient). This was offered monthly until 12 months after either elimination of symptoms or plateau of improvement. Athletes were not required to receive dextrose injection if they were satisfied with their status at 3 months. The athletes were seen in clinic at 6 months and 1 year to be sure that those that reported no pain or stiffness were indeed asymptomatic when performing a single leg squat, and to update contact information and minimize potential for data dropout. - *General advice*: All athletes were given pictorial sheets of gently progressive hamstring stretching and quads strengthening exercises.   Dextrose and local anaesthetic   - *Injection*: 1% lidocaine and 12.5% dextrose - *General treatment after injections*: Acetaminophen was advised if neededfor postinjection discomfort. Athletes were advised not to run or kick for 1week after the first injection, and to run as tolerated after the first week.They were advised not to run or kick for 3 days after both the second and any subsequent injections. Usually,they started playing sports with com-petition if doing well after the second injection. Note that many athletes with an NPPS score of 3 were used to play-ing with pain; these athletes were encouraged to engage in a sporting activity only if the activity was not accompanied or followed by pain during the period of treatment.As an incentive for study participation,and to potentially avoid sports drop-out, all study participants that did not reach an NPPS score of 0 could choose to receive dextrose injection after 3 months (the point at which the actual injectant was revealed to the treating physician and patient). This was offered monthly until 12 months after either elimination of symptoms or plateau of improvement. Athletes were not required to receive dextrose injection if they were satisfied with their status at 3 months. The athletes were seen in clinic at 6 months and 1 year to be sure that those that reported no pain or stiffness were indeed asymptomatic when performing a single leg squat, and to update contact information and minimize potential for data dropout. - *General advice*: All athletes were given pictorial sheets of gently progressive hamstring stretching and quads strengthening exercises.   Usual care   - *Injection*: None. - *General treatment after injections*: None. - *General advice*: Those in the usual care group met with a physical therapist who instructed them individually in the stretching and exercise method, and provided a video. Then, those in the usual care groups returned at least once for 1-on-1 confirmation of proper exercise performance and to encourage compliance. |
| **Outcomes** | *NPSS*   - **Outcome type**: ContinuousOutcome - **Direction**: Lower is better - **Data value**: Endpoint   *NPSS score 4*   - **Outcome type**: DichotomousOutcome - **Reporting**: Fully reported - **Data value**: Endpoint   *NPSS score of 0*   - **Outcome type**: DichotomousOutcome - **Reporting**: Fully reported - **Data value**: Endpoint |
| **Identification** | **Sponsorship source:** None.  **Country:** Argentina  **Setting:** Athletes screened for anterior knee pain  **Comments:** -  **Authors name:** Gaston Topol  **Institution:** Departments of Physical Medicine and Rehabilitation and Orthopedics  **Email:** deanreevesmd@gmail.com  **Address:** Hospital Provincial de Rosario, Rosario, Argentina |
| **Notes** | *Benjamin Dean* on 27/12/2017 09:44 **Population** No baseline charactistics of different groups detailed. |

**Risk of bias table**

| **Bias** | **Authors' judgement** | **Support for judgement** |
| --- | --- | --- |
| Random sequence generation (selection bias) |  | Quote: "sent, a random numbers table was used for assignment to supervised usual care or to an injection solution group blinded to the subject, guardian, and the treating/evaluating physician." |
| Allocation concealment (selection bias) |  | Judgement Comment: a random number table was used |
| Blinding of participants and personnel (performance bias) |  |  |
| Blinding of outcome assessment (detection bias) |  | Quote: "The solution for each visit was prepared by the physician who as- signed the patient, and was prepared in a manner blinded to the patient and the treating/evaluating physician." |
| Blinding of outcome assessment (detection bias) |  | Quote: "The solution for each visit was prepared by the physician who as- signed the patient, and was prepared in a manner blinded to the patient and the treating/evaluating physician." |
| Incomplete outcome data (attrition bias) |  |  |
| Selective reporting (reporting bias) |  | Judgement Comment: No protocol specified with primary outcome. |
| Other bias |  |  |

**Trail 1988**

| **Methods** | **Study design:** Retrospective cohort study  **Study grouping:** Parallel group |
| --- | --- |
| **Participants** | **Baseline Characteristics**  Conservative   - *age range*: 10 to 17 - *age average*: 12 years 7 months - *duration of symptoms range*: 9 months to 4 years - *average (SEM) duration of symptoms*: 1.79 (0.24)   Surgical   - *age range*: 11 to 17 - *age average*: 13 years 9 months - *duration of symptoms range*: 3 months to 4 years - *average (SEM) duration of symptoms*: 1.34 (0.14)   Overall   - *age range*: 10 to 17 - *age average*: - - *duration of symptoms range*: 3 months to 4 years - *average (SEM) duration of symptoms*: -   **Included criteria:** Osgood-Schlatter disease , but specific criteria not specified  **Excluded criteria:** Unknown  **Pretreatment:** Conservatively treated compared with surgical treatment  **conservative:** -  **surgical:** - |
| **Interventions** | **Intervention Characteristics**  Conservative   - *Advice*: Reduction of activity and avoidance of sports - *Surgery*: None - *Casting*: 3 treated with period in extension cast - *Physiotherapy*: 3 had physiotherapy with ice packs and ultrasound therapy - *Injections*: 4 injected with local anaesthetic and steroid   Surgical   - *Advice*: Reduction of activity and avoidance of sports - *Surgery*: Tibial sequestrectomy - *Casting*: 4 treated with period in extension cast - *Physiotherapy*: 4 had physiotherapy with ice packs and ultrasound therapy - *Injections*: 9 injected with local anaesthetic and steroid |
| **Outcomes** | *Asymptomatic*   - **Outcome type**: DichotomousOutcome - **Reporting**: Fully reported - **Data value**: Endpoint   *Complication rate*   - **Outcome type**: AdverseEvent - **Reporting**: Fully reported - **Direction**: Lower is better - **Data value**: Endpoint |
| **Identification** | **Sponsorship source:** None  **Country:** England  **Setting:** Royal Manchester Children's Hospital  **Comments:** -  **Authors name:** I A Trail  **Institution:** Royal Manchester Children's Hospital  **Email:** No email listed  **Address:** 20 Ridingfold Lane, Manchester, M28 4PZ |
| **Notes** | *Benjamin Dean* on 27/12/2017 01:22 **Outcomes** Complications in: operated group were 55% (consisted of 18 with bony prominence, 1 with reduction in range of movement, 1 with recurvatum, 1 with wound dehiscence, 1 with infection, 3 with lateral scar numbness) non operated group (6 with bony prominence) |

**Risk of bias table**

| **Bias** | **Authors' judgement** | **Support for judgement** |
| --- | --- | --- |
| Random sequence generation (selection bias) |  | Judgement Comment: Not randomised trial. |
| Allocation concealment (selection bias) |  | Judgement Comment: Not randomised trial. |
| Blinding of participants and personnel (performance bias) |  |  |
| Blinding of outcome assessment (detection bias) |  | Judgement Comment: Outcomes assessed by treating clinicians. |
| Incomplete outcome data (attrition bias) |  | Judgement Comment: Retrospective and hence high risk of selective reporting. |
| Selective reporting (reporting bias) |  | Judgement Comment: No protocol defined. No primary outcome defined. |
| Other bias |  | Judgement Comment: Study design highly prone to bias. |

**vanArk 2016**

| **Methods** |  |
| --- | --- |
| **Participants** |  |
| **Interventions** |  |
| **Outcomes** |  |
| **Identification** |  |
| **Notes** |  |

**Risk of bias table**

| **Bias** | **Authors' judgement** | **Support for judgement** |
| --- | --- | --- |
| Random sequence generation (selection bias) |  | Quote: "Participants were randomised to an exercise program by the draw of a sealed opaque envelope from 40 identical envelopes that were randomised using a randomisation table created by com- puter software (20 in each group)." |
| Allocation concealment (selection bias) |  |  |
| Blinding of participants and personnel (performance bias) |  |  |
| Blinding of outcome assessment (detection bias) |  | Judgement Comment: Not described if assessors were blinded. |
| Incomplete outcome data (attrition bias) |  | Judgement Comment: 9 participants' data not included (20/29) |
| Selective reporting (reporting bias) |  | Quote: "Median pain scores improved signiﬁcantly over the 4-week intervention period in both the isometric group (Z = −2.527, p = 0.012, r = −0.63) and isotonic group (Z = −2.952, p = 0.003, r = −0.63). There was no signiﬁcant difference in NRS pain score change (U = 29.0, p = 0.208, r = 0.29) between the isometric group (median (IQR), 2.5 (1–4.5)) and isotonic group (median (IQR), 3.0 (2–6))."  Quote: "The primary outcome measure was pain during a single leg decline squat (SLDS) scored on a numeric rating scale (NRS) (0–10), which is a provocative clinical test to monitor tendon pain."  Judgement Comment: Protocol and specified primary outcome |
| Other bias |  |  |

**Wang 2007**

| **Methods** | **Study design:** Randomized controlled trial  **Study grouping:** Parallel group |
| --- | --- |
| **Participants** | **Baseline Characteristics**  Control group   - *Number of patients*: 23 - *Number of knees*: 24 - *Average age (SD)*: 30.2 (10.4) - *Age range*: 16 to 46 - *Average duration of symptoms (SD)*: 11.3 (10.9)   Extracorporeal shockwave treatment   - *Number of patients*: 27 - *Number of knees*: 30 - *Average age (SD)*: 29.4 (10.5) - *Age range*: 16 to 47 - *Average duration of symptoms (SD)*: 16.2 (17.2)   Overall   - *Number of patients*: - - *Number of knees*: - - *Average age (SD)*: - - *Age range*: - - *Average duration of symptoms (SD)*: -   **Included criteria:** 1. Patients with a diagnosis of chronic patellar tendinopathy established by medical history and physical examination2. Patients who experienced pain of 5.0 or greater on a 0-to-10 visual analog scale while walking up and down stairs3. Patients who understood and complied with the nature of the study participation4. Patients who were 21 years and older and skeletally matured5. Patients who were physically and mentally competent to sign the informed consent form6. Patients who were in good general health  **Excluded criteria:** 1. Patients who received a cortisone injection within 6 weeks2. Patients on immunosuppressant agents and/or corticosteroid within 6 months3. Patients with diabetes mellitus, occlusive vascular disease, collagen disease, osteoarthritis or rheumatoid arthritis, coagulopathy, or infection4. Patients with radiographic fractures around the knee5. Patients with cardiac arrhythmia or cardiac pacemaker6. Patients who were pregnant  **Pretreatment:** - |
| **Interventions** | **Intervention Characteristics**  Control group   - *Shockwave treatment*: None - *General treatment*: Patients in the control group were treated with conservative treatments including NSAIDs, physiotherapy, an exercise program, the use of a knee strap, and modification of activity levels. Physiotherapy modalities included hot and cold packs, phonophoresis, and friction massage. Theexercise program included a primarily eccentric stretching exercise of the patellar tendon and strengthening exercises of the quadriceps and hamstrings. Most patients started out with the administration of NSAIDs. A Cox-2 specific inhibitor (celecoxib) was prescribed to patients who developed allergic reaction or gastrointestinal toxicity t oNSAIDs. Patients were then treated with multiple modalities when they failed to respond adequately to treatment with a single modality. Nearly all patients eventually received multiple modalities during the course of treatment. None received local cortisone injection. - *Second shockwave treatment*: None   Extracorporeal shockwave treatment   - *Shockwave treatment*: Patients in the study group received shockwave treatment as outpatients with no local or regional anesthesia. The source of shockwave was from an OssaTron (High MedicalTechnology, Kreuzlingen, Switzerland). Each knee was treated with 1500 impulses of shockwave at 14 KV (equiva-lent to 0.18 mJ/mm² energy flux density) in a single session.The dosage was chosen based on our previous experience in shockwave application for other tendinopathies.The point of maximal tenderness was elicited by palpation,and the location of the lesion was focused with the laser control guide of the device. The depth of treatment was estimated clinically and confirmed with an ultrasound guide. Surgical lubricant was applied to the skin in contact with the shock-wave tube. Treatment began with slow frequency at 1 impulse of shock per second and gradually increased to 2 shocks per second as the patient could tolerate the procedure. Immediately after treatment, the area was inspected forswelling, ecchymosis, and hematoma. - *General treatment*: Postoperative management included ice pack to the treatment site and a prescription of nonnarcotic analgesic, such as acetaminophen.Patients were allowed to resume light activity; however,heavy activities including sports were not permitted for 4 to 6 weeks. - *Second shockwave treatment*: Three patients (4 knees) also received a second treatmentbecause of either inadequate response or recurrent symptoms 4 to 6 weeks after the first treatment. Inadequate response was defined when the patient showed less than 50% improvement and experienced pain at 5.0 or greater on a 0-to-10 visual analog scale (VAS) on stairs and palpation.The dosage of the second shockwave application was the same as the first treatment. |
| **Outcomes** | *Pain scores (VAS)*   - **Outcome type**: ContinuousOutcome - **Data value**: Endpoint   *VISA scores*   - **Outcome type**: ContinuousOutcome - **Data value**: Endpoint   *Range of knee movement*   - **Outcome type**: ContinuousOutcome - **Data value**: Endpoint   *Functional improvement %*   - **Outcome type**: ContinuousOutcome - **Data value**: Endpoint |
| **Identification** | **Sponsorship source:** The funding source was from Chang Gung Research Fund(CMRP 8015) and National Health Research Institute(NHRI-EX95-9423EP)  **Country:** Taiwan  **Setting:** Orthopaedic out patient clinic  **Comments:** Note author has responded to state no patients took part aged under 18 years of age  **Authors name:** Ching-Jen Wang  **Institution:** Department of Orthopaedic Surgery  **Email:** w281211@adm.cgmh.org.tw  **Address:** Chang Gung Memorial Hospital, Chang Gung University College of Medicine, 123 Ta-Pei Road, Niao-Sung Hsiang,Kaohsiung, Taiwan |
| **Notes** | *Benjamin Dean* on 25/12/2017 01:36 **Included** note sent 24/12/17  *Benjamin Dean* on 28/12/2017 20:43 **Included** no patients 18 according to author response |

**Risk of bias table**

| **Bias** | **Authors' judgement** | **Support for judgement** |
| --- | --- | --- |
| Random sequence generation (selection bias) |  | Unclear |
| Allocation concealment (selection bias) |  | Judgement Comment: Not described. |
| Blinding of participants and personnel (performance bias) |  | Judgement Comment: Participants not blinded. |
| Blinding of outcome assessment (detection bias) |  | Quote: "The remaining evaluations were performed in person by an independent examiner blinded to the nature of the study protocol."  Judgement Comment: Not mentioned if evaluations performed by person who was blinded to the actual treatment (different to 'nature of study protocol') |
| Incomplete outcome data (attrition bias) |  | Quote: "During the course of treatment, 3 patients (4 knees) were lost to follow-up and were excluded from the study." |
| Selective reporting (reporting bias) |  | Quote: "the 2- to 3-year follow-up, the overall results for the study group were 43% excellent, 47% good, 10% fair, and none poor. For the control group, the results were none excellent, 50% good, 25% fair, and 25% poor. The mean Victorian Institute of Sports Assessment scores were 42.57 ± 10.22 and 39.25 ± 10.85, respectively, before treatment (P = .129) and 92.0 ± 10.17 and 41.04 ± 10.96, respectively, after treatment (P < .001). Satisfactory results were observed in 90% of the study group versus 50% of the control group (P < .001). Recurrence of symptoms occurred in 13% of the study group and 50% of the control group (P = .014). Ultrasonographic examination showed a significant increase in the vascularity of the patellar tendon and a trend of reduction in the patellar tendon thickness after shockwave treatment compared with conservative treatments."  Judgement Comment: Multiple outcomes, no primary outcome specified, and no protocol cited. |
| Other bias |  | Quote: "OssaTron (High Medical Technology, Kreuzlingen, Switzerland)."  Judgement Comment: Financial relationship with company not described. |

**Willberg 2011**

| **Methods** | **Study design:** Randomized controlled trial  **Study grouping:** Parallel group |
| --- | --- |
| **Participants** | **Baseline Characteristics**  Sclerosing polidocanol injections   - *age in years - mean (SD)*: 27 (7.6) - *duration of symptoms, months - mean (SD)*: 20 (10.4) - *VAS at rest at assessment - mean (SD)*: 37.8 (24.9) - *VAS at activity at assessment - mean (SAD)*: 69.0 (17.3)   Surgery - arthoscopic shaving   - *age in years - mean (SD)*: 26.6 (7.6) - *duration of symptoms, months - mean (SD)*: 23.8 (15.5) - *VAS at rest at assessment - mean (SD)*: 44.6 (28.4) - *VAS at activity at assessment - mean (SAD)*: 76.5 (13.6)   Overall   - *age in years - mean (SD)*: - - *duration of symptoms, months - mean (SD)*: - - *VAS at rest at assessment - mean (SD)*: - - *VAS at activity at assessment - mean (SAD)*: -   **Included criteria:** Patellar tendinopathy as confirmed by ultrasound and colour Doppler showing structural tendon changes and high blood flow on the dorsal side of the proximal patellar tendon  **Excluded criteria:** -  **Pretreatment:** - |
| **Interventions** | **Intervention Characteristics**  Sclerosing polidocanol injections   - *Surgery*: No surgery. - *Injection*: The injection was performed with a 0.7×50 mm needle connected to a 2 ml syringe. The same experienced ultra-sonographer performed all ultrasound and colour Doppler examinations and injections. The injection was performed dynamically, with the aid of real-time grey-scale ultrasound and the colour Doppler technique, to inject at the target vessels. Small volumes (0.1–0.2 ml, maximum 2 ml) of the substance were injected into the regions with local neovas-cularisation/high blood fl ow dorsal to the proximal patellar tendon. A dressing was applied for 24 h. A maximum of three injection treatments (at least 6 weeks in between) were given before evaluation. - *General advice and rehab*: The patients in both groups were allowed full weightbearing walking immediately after the treatment. Two weeks after treatment the patients were told gradually to increase the patellar tendon load up to full loading. There was no specific rehabilitation protocol, or set time frames, preceding full ten-don loading activity.   Surgery - arthoscopic shaving   - *Surgery*: Arthroscopy was performed under local anaesthesia. The patients were in a supine position with straight knee and relaxed quadriceps. We used standard anteromedial and anterolateral portals, and a pressure controlled pump. No tourniquet was used. Ultrasound and colour Doppler was used preoperatively. Initially, a standard arthroscopic eval-uation of the whole knee joint was performed. Then the patellar tendon insertion into the patella was identified. For shaving, a 4.5-mm full radius blade shaver was used. Simultaneous ultrasound examination (longitudinal and transversal views) guided the procedure. Careful shaving, aiming to destroy only the region with high blood flow (neovessels) and nerves adjacent to the tendinosis changes on the dorsal side of the tendon, was done (ie, separat-ing the Hoffa fat pad from the patellar tendon). No tendon tissue was resected and the Hoffa fat pad was saved. The portals were closed with a tape, and a bandage was used for 24 h. - *Injection*: No injections. - *General advice and rehab*: The patients in both groups were allowed full weightbearing walking immediately after the treatment. Two weeks after treatment the patients were told gradually to increase the patellar tendon load up to full loading. There was no specific rehabilitation protocol, or set time frames, preceding full ten-don loading activity. |
| **Outcomes** | *VAS at rest*   - **Outcome type**: ContinuousOutcome - **Direction**: Lower is better - **Data value**: Endpoint   *VAS at activity*   - **Outcome type**: ContinuousOutcome - **Direction**: Lower is better - **Data value**: Endpoint   *Satisfaction with result*   - **Outcome type**: ContinuousOutcome - **Direction**: Higher is better - **Data value**: Endpoint |
| **Identification** | **Sponsorship source:** None  **Country:** Sweden  **Setting:** Capio Artro Clinic  **Comments:** -  **Authors name:** Lotta Willberg  **Institution:** Stockholm Sports Trama Research Centre, Karolinska Institutet  **Email:** l o t t a . w i l l b e r g @ c a p i o . s e  **Address:** Stockholm, Sweden |
| **Notes** | *Benjamin Dean* on 25/12/2017 00:36 **Included** author emailed for data 24/12/17  *Benjamin Dean* on 18/01/2018 21:42 **Included** email hakan alfredson sent |

**Risk of bias table**

| **Bias** | **Authors' judgement** | **Support for judgement** |
| --- | --- | --- |
| Random sequence generation (selection bias) |  | Quote: "groups were not too small. <b>Randomisation After receiving oral and written information, from an inde- pendent assistant the patients selected an envelope (52 opaque envelopes), allocating themselves to either treatment with</b> sclerosing polidocanol injections (10 mg/ml)" |
| Allocation concealment (selection bias) |  | Judgement Comment: opaque envelopes |
| Blinding of participants and personnel (performance bias) |  |  |
| Blinding of outcome assessment (detection bias) |  |  |
| Incomplete outcome data (attrition bias) |  | Judgement Comment: No mention in text or tables as to how many were followed up. |
| Selective reporting (reporting bias) |  | Quote: "and 12 months after treatment. <b>Outcomes The patients scored the level of patellar tendon pain during their speciﬁ c sport activity, and at rest, on a 100-mm long visual analogue scale (VAS). The amount of pain was recorded from 0 to 100 mm, where no pain was recorded as 0 and severe pain as 100. Self-reported patient satisfaction with the result of the treat- ment (0–100% satisfaction), was also scored by the patients on a 100-mm long scale.</b> B A Figure 1 Grey-scale"  Judgement Comment: No clearly defined primary outcome. Protocol not detailed or cited. |
| Other bias |  |  |

**Footnotes**
