# Supplementary figures and images for: Therapeutic interventions in children and adolescents with patellar tendon related pain: a systematic review
Source: BMJ Open Sport Exerc Med. 2018 Aug 13;4(1):e000383. doi: 10.1136/bmjsem-2018-000383 (PMC6109948; doi:10.1136/bmjsem-2018-000383)

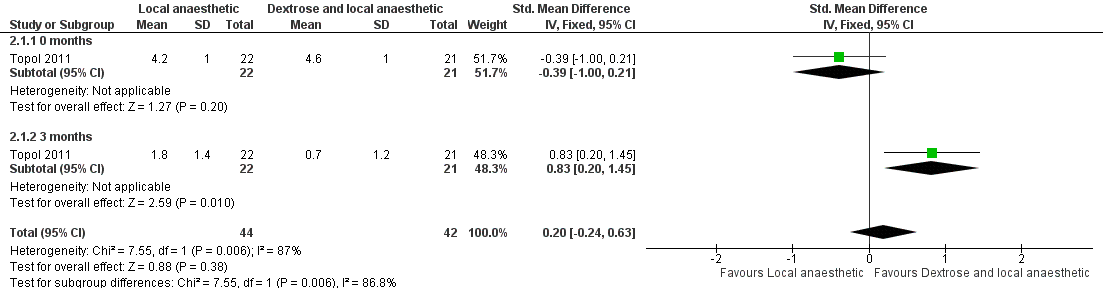

Supplement: Supplementary data [file bmjsem-2018-000383supp003.png]

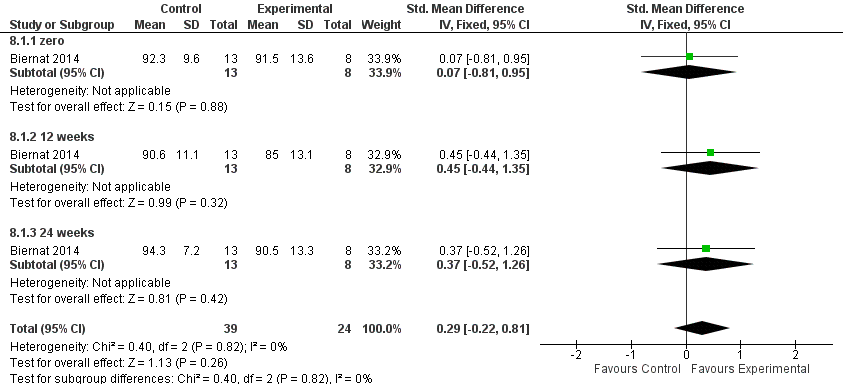

Supplement: Supplementary data [file bmjsem-2018-000383supp004.png]

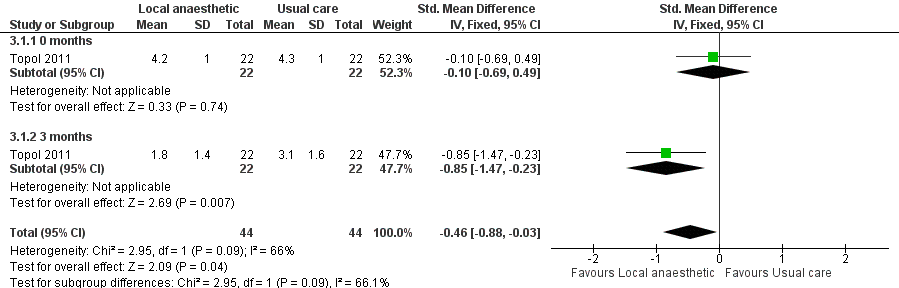

Supplement: Supplementary data [file bmjsem-2018-000383supp005.png]

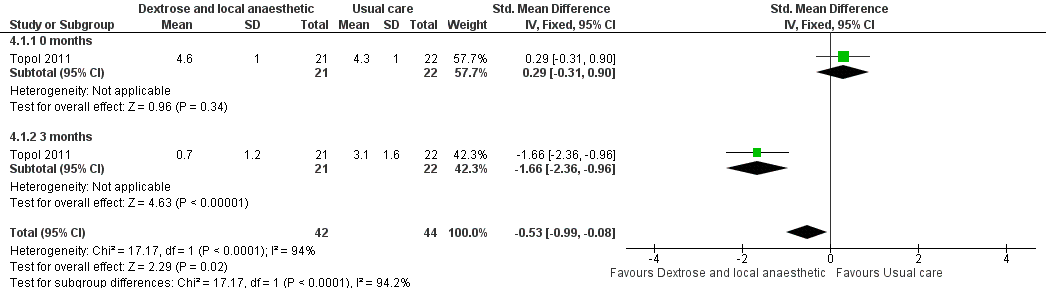

Supplement: Supplementary data [file bmjsem-2018-000383supp006.png]

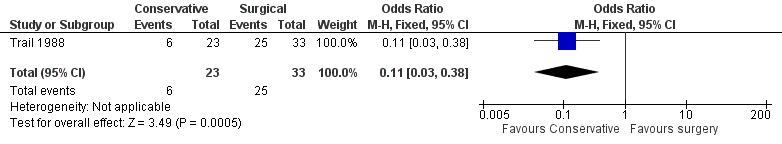

Supplement: Supplementary data [file bmjsem-2018-000383supp007.png]

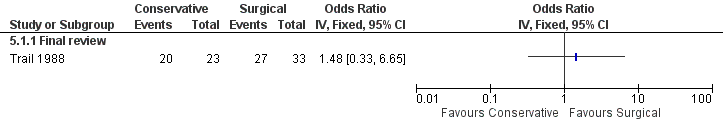

Supplement: Supplementary data [file bmjsem-2018-000383supp008.png]
